# Chirality Evolution of Supramolecular Helices by Electron Transfer Assisted Secondary Nucleation

**DOI:** 10.1002/advs.202408499

**Published:** 2024-12-16

**Authors:** Laiben Gao, Xiaoqiu Dou, Chao Xing, Kaikai Yang, Changli Zhao, Chuanliang Feng

**Affiliations:** ^1^ State Key Lab of Metal Matrix Composites School of Materials Science and Engineering Shanghai Key Laboratory for Molecular Engineering of Chiral Drugs Shanghai Jiao Tong University Shanghai 200230 P. R. China

**Keywords:** chirality evolution, electron transfer, helical nanofibers, secondary nucleation, supramolecular assembly

## Abstract

Chirality evolution is ubiquitous and important in nature, but achieving it in artificial systems is still challenging. Herein, the chirality evolution of supramolecular helices based on l‐phenylalanine derivative (LPF) and naphthylamide derivate (NDIAPY) is achieved by the strategy of electron transfer (ET) assisted secondary nucleation. ET from LPF to NDIAPY can be triggered by 5 s UV irradiation on left‐handed LPF‐NDIAPY co‐assemblies, leading to NDIAPY radical anions and partial disassembly of the helices. Meanwhile, spontaneous reversion of radical anions into monomers occurs upon removal of UV light, and the surface of residual co‐assemblies can accelerate the reversion process. This surface accelerated reversion of ET further facilitates the secondary nucleation‐elongation events, giving rise to the formation of scale‐amplified and g vale‐increased left‐handed helices. Meanwhile, chirality evolution controlled by ET assisted secondary nucleation process can be also realized by adding the prepared LPF‐NDIAPY co‐assemblies into the total ET system. This study may provide a useful approach to constructing and modulating diverse chiral structures by manipulating the secondary nucleation process.

## Graphical abstract

Chirality evolution is ubiquitous and important in nature, a strategy to control the chirality evolution (Asymmetry amplification) is developed of supramolecular helices by electron transfer (ET) assisted secondary nucleation. For the electron transferred system, the reversion of ET can be accelerated by the surface of assemblies, which further facilitates the secondary nucleation‐elongation events, giving rise to the formation of scale‐amplified and g vale‐increased left‐handed helices.

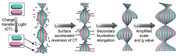

## 1 Introduction

The evolution of chirality is ubiquitous in natural organisms and plays a key role in determining the physiological function,^[^ ^1^, ^2^, ^3^, ^4^, ^5^, ^6^ ^]^ in which secondary nucleation is usually involved (e.g. protein aggregation).^[^ ^7^, ^8^ ^]^ To date, diverse chirality evolutions (e.g. chirality induction, transfer, inversion, and amplification) have been manipulated by varying parameters such as temperature,^[^ ^9^, ^10^, ^11^ ^]^ solvent,^[^ ^12^, ^13^, ^14^ ^]^ cooling rate,^[^ ^15^, ^16^, ^17^, ^18^, ^19^ ^]^ pH,^[^ ^20^ ^]^ during the preparation process, or by introducing the additives.^[^ ^21^, ^22^, ^23^, ^24^, ^25^, ^26^ ^]^ For these processes, the stacking modes of building blocks are altered at the primary nucleation‐elongation process, then leading to diverse chiral structures.^[^ ^27^, ^28^, ^29^, ^30^, ^31^, ^32^ ^]^ However, secondary nucleation mediated chirality evolution is still missing, leading to the complexity and controllability of chiral structures in man‐made systems being far behind natural biological systems, since secondary nucleation and subsequent elongation offer rich opportunities for chirality transformation. Thus, it is highly expected to realize artificial regulation of chiral architectures by applying secondary nucleation and unraveling the regulation mechanism.

Supramolecular assembly has provided an efficient way to produce different chiral structures,^[^ ^33^, ^34^, ^35^, ^36^, ^37^, ^38^, ^39^, ^40^, ^41^ ^]^ due to its flexible intermolecular interactions.^[^ ^42^, ^43^, ^44^, ^45^, ^46^, ^47^, ^48^, ^49^, ^50^, ^51^ ^]^ Nevertheless, secondary nucleation regulated chiral assembly, and its structural evolution remain scarce because of the intricate relationship between secondary nucleation and chirality transformation. Despite the great challenge in secondary nucleation induced chirality evolution, surface‐catalyzed secondary nucleation has been exploited to finely regulate various other architectures.^[^ ^52^, ^53^, ^54^, ^55^ ^]^ For instance, the elegant nanostructures that cannot be achieved by primary nucleation are well fabricated by secondary nucleation (such as self‐assembled poly‐catenanes prepared by Yagai et al.,^[^ ^56^ ^]^ double‐stranded Archimedean spirals, concentric toroid and 2D block self‐assembled polymers reported by Sugiyasu and co‐workers,^[^ ^57^, ^58^ ^]^ 3D spherical spherulites and scarf‐like structures created by Rao et al.^[^ ^59^ ^]^). Meanwhile, George group reported serials of work on secondary nucleation events in supramolecular assembly including stereoselective secondary nucleation,^[^ ^60^ ^]^ secondary nucleation‐triggered bundling of supramolecular nanofibers^[^ ^61^ ^]^ as well as enhancement of structural complexity,^[^ ^62^ ^]^ which greatly accelerate the understanding and application of secondary nucleation in creating novel supramolecular architectures. These achievements constitute a foundation for the research of chirality evolution regulated by secondary nucleation.

**Figure 1:**
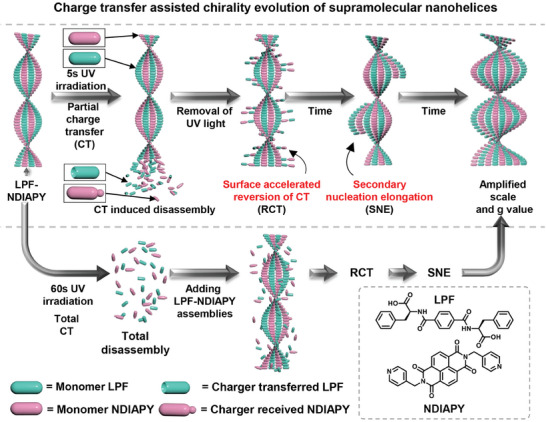
Schematic illustration of asymmetric amplification of supramolecular helices by ET assisted secondary nucleation. For the left‐handed helices constructed by the co‐assembly of LPF and NDIAPY, the incomplete ET from LPF to NDIAPY can be triggered by 5 s UV irradiation, resulting in the formation of NDIAPY radical anions and partial disassembly of nanohelices. After removing UV light, spontaneous reversion of radical anions into monomers occurs, then subsequent reassembly is triggered, giving rise to the nanohelices with amplification in scale as well as g value because of surface accelerated recovery of ET and surface catalyzed secondary nucleation.

Herein, the chirality evolution of supramolecular helices based on l‐phenylalanine derivatives (LPF) and naphthylamide derivates (NDIAPY) is successfully achieved by electron transfer (ET) assisted secondary nucleation (**Figure** **1**). ET from LPF to NDIAPY is triggered by 5 s UV irradiation on the left‐handed helix of LPF‐NDIAPY co‐assemblies, leading to the formation of NDIAPY radical anions and partial disassembly of the helices. Spontaneous reversion of radical anions into monomers occurs upon removal of UV light, and the surface of residual co‐assemblies can accelerate the reversion process. Subsequently, surface accelerated reversion of ET facilitates the secondary nucleation‐elongation events, giving rise to the formation of scale‐amplified and g vale‐increased left‐handed helices. Meanwhile, adding the prepared LPF‐NDIAPY co‐assemblies into the total ET system can also result in this chirality evolution via ET assisted secondary nucleation process. This strategy of ET assisted secondary nucleation could not only regulate other chirality evolutions (e.g. chirality inversion, chiral morphological transformation) but also conveniently produce exquisite structures in an artificial system.

## 2 Results and Discussion

### 2.1 Photo‐Induced Chirality Evolution of Supramolecular Helices

The l‐phenylalanine derivative (LPF) and naphthylamide derivative (NDIAPY) were synthesized and confirmed by ^1^H/^13^C nuclear magnetic resonance (^1^H/^13^C NMR) spectroscopy and high‐resolution mass spectrometry (Figures S1–S6, Supporting Information). The enantiomeric purity and absolute configuration of LPF and NDIAPY were determined by chiral high‐performance liquid chromatography (Chiral HPLC) (Figures S7–S9, Supporting Information) and single crystal (CCDC number: 2282095 for LPF, 2246812 for NDIAPY) (Figures S10 and S11, Supporting Information), respectively.

**Figure 2:**
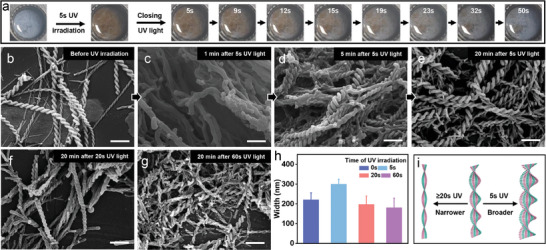
a) The color change of LPF‐NDIAPY co‐assemblies before and after removing 5 s UV irradiation. LPF: 2 mM, NDIAPY: 2 mM, room temperature. b) SEM images of LPF‐NDIAPY co‐assemblies before UV irradiation. Scale bar: 1 µm. c–e) Time‐dependent morphology evolution of LPF‐NDIAPY after 5 s UV irradiation. (c) after 1 min, (d) after 5 min, (e) after 20 min. Scale bar: 1 µm. f,g) SEM images of (f) 20s and (g) 60 s UV‐treated LPF‐NDIAPY after removing the irradiation for 20 min. Scale bar: 1 µm. h) The width of LPF‐NDIAPY assemblies after 0, 5, 20, 60 s UV irradiation. i) Schematic illustration of width changes upon different UV‐treated times.

The stock solution of LPF and NDIAPY was prepared by dissolving them in a hexafluoroisopropanol (HFIP) solution (Concentration: LPF: 20 mM, NDIAPY: 20 mM). The monomeric state of LPF and NDIAPY in stock solution was confirmed by 1) the consistent UV peaks between the stock solution with or without 10 folded dilution (Figure S12, Supporting Information), 2) the accordant UV spectra between the spectra of stock solution and the simple sum of monomeric LPF and NDIAPY spectra (Figure S13, Supporting Information). The LPF‐NDIAPY co‐assembly was triggered by adding 900 µL H_2_O into 100 µL hexafluoroisopropanol (HFIP) stock solution of LPF and NDIAPY (final concentration: LPF: 2 mM, NDIAPY: 2 mM). After co‐assembly, a UV peak at 379 nm observed from the stock solution shifted to 382 nm (Figure S13, Supporting Information), further indicating the monomeric state of LPF and NDIAPY in stock solution and H_2_O triggered assembly. A transient UV irradiation (5 s, 365 nm, 10W) on LPF‐NDIAPY co‐assembly gave rise to the color transition from white to dark brown (**Figure** **2**a) (This color change was ascribed to electron transfer from LPF to NDIAPY, vide infra), and removing UV irradiation resulted in the color recovery to white after ≈50 s. Such reversible light‐responsive behavior could be in situ and repeated for at least 5 cycles (Figure S14, Supporting Information). UV irradiation can also result in the color change for LPF‐NDIAPY in DMF/ H_2_O (1:9, v/v) (Figure S15a,b, Supporting Information), indicating that HFIP was not the necessary factor for the electron transfer process. Besides, for LPF or NDIAPY in HFIP/H_2_O (1:9, v/v), no color change is observed after UV irradiation (Figure S15c,d, Supporting Information), indicating that the electron transfer is dependent on the coexistence of LPF and NDIAPY. Furthermore, the influence of dissolved oxygen on this electron transfer was studied by controlling the oxygen concentration in water via bubbling nitrogen gas (This water is labeled as H_2_O_nitrogen_) (Figure S16, Supporting Information). When the LPF‐NDIAPY in HFIP/ H_2_O_nitrogen_ (1:9, v/v) suffers 5 s UV irradiation, the color transition from dark brown to white was also accomplished after ≈50 s, which is consistent with that of LPF‐NDIAPY in HFIP/ H_2_O (1:9, v/v). It indicated that the dissolved oxygen is a negligible factor in the electron transfer process.

Scanning electron microscopy (SEM) revealed that the left‐handed helical structures with a width of ≈224 ± 32 nm and helical pitch of ≈630 nm were formed before UV irradiation (Figure 2b). 5 s UV irradiation treated sample exhibited chiral morphology evolution upon removing UV light (after ≈1 min: columnar structures, Figure 2c; after ≈5 min: left‐handed helix, Figure 2d; after ≈20 min: left‐handed helix with increased width of 303 ± 22 nm, Figure 2e), indicating the width amplification after 5 s UV irradiation. With tuning UV irradiation time, this chirality evolution was further investigated. 20 s UV irradiated sample first presented mixtures of columnar and spheric structures after ≈1 min and then transformed into left‐handed helix with width of 200 ± 40 nm (Figure 2f; Figures S17 and S18, Supporting Information), and 60 s irradiation gave rise to exclusive spheric structures at ≈1 min and left‐handed helix with width of 184 ± 45 nm after 20 min (Figure 2g, h; Figures S17 and S18, Supporting Information). These results indicated that sufficient UV irradiation led to the total disassembly of LPF‐NDIAPY fibers. Furthermore, the width changes after different UV irradiation indicated that only a specific 5 s UV irradiation time could facilitate the reorganization of LPF‐NDIAPY assemblies into scale‐amplified chiral structures, but excessive irradiation time hindered this process (Figure 2i).

**Figure 3:**
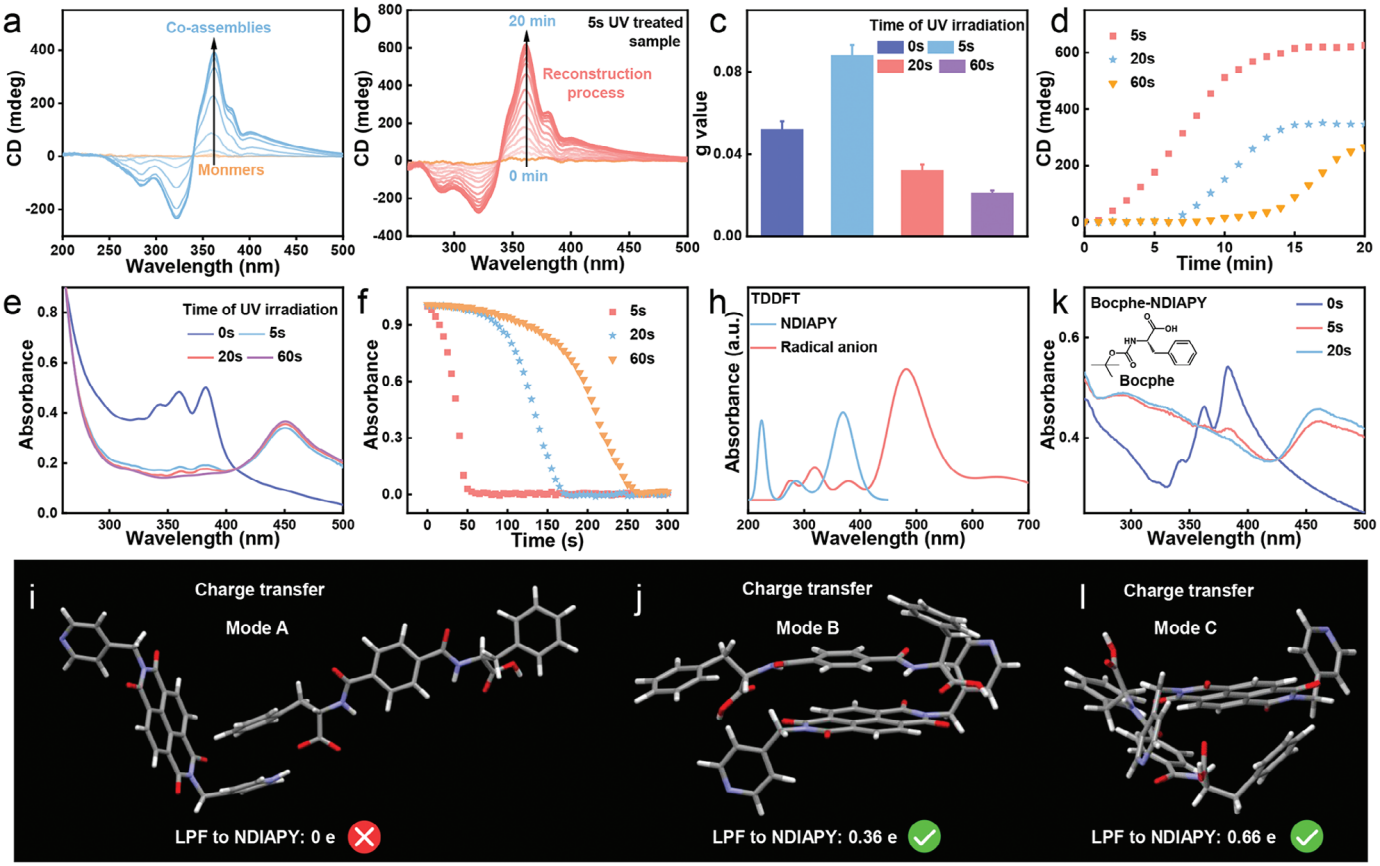
a) Time‐dependent CD spectra of LPF‐NDIAPY. b) Time‐dependent CD spectra of LPF‐NDIAPY after 5 s UV irradiation. c) The g values (361 nm) of 0s/5 s/20 s/60 s treated LPF‐NDIAPY. d) Time‐dependent CD signals at 361 nm for the reassembly process after 5 s/20 s/60 s UV irradiation. e) UV spectra of LPF‐NDIAPY after 5 s/20 s/60 s UV irradiation. f) Time‐dependent UV peak at 382 nm for LPF‐NDIAPY after 5 s/20 s/60 UV irradiation. h) The calculated UV spectra of NDIAPY and its radical anion by TDDFT calculations. k) UV spectra of Bocphe‐NDIAPY after 0s/5 s/20 s UV irradiation. i,j,l) The singlet excited‐state of LPF‐NDIAPY optimized dimer based on (i) hydrogen bond between carboxylic group and pyridine, (j) close contact between phenyl core and NDI core and (l) close contact between Phe group and NDI core.

Circular dichroism (CD) spectroscopy was employed to investigate the optical activities of these chiral structures. The HFIP stock solution of LPF and NDIAPY was CD silent, corresponding to the monomer state. With the addition of H_2_O into the stock solution to trigger co‐assembly, a negative peak at 285 nm and a positive peak at 361 nm was observed, and the intensity of both peaks gradually increased with extending the assembled time (**Figure** **3**a; Figure S19, Supporting Information). Meanwhile, no peak was observed from linear dichroism (LD) spectra (Figure S20, Supporting Information), implying that the macroscopic alignment was absent and the CD spectra of LPF‐NDIAPY do not include contributions from LD spectra. However, upon 5 s UV irradiation, both CD peaks at 285 and 361 nm immediately disappeared (Figure 3b, 0 min), indicating UV‐induced disassembly. These disassembled LPF‐NDIAPY could be reconstructed as evidenced by the gradual increase of CD intensity at 285 and 361 nm within 20 min after removing UV light (Figure 3b). A similar reconstruction process was also detected for 20 and 60 s treated samples (Figure S19, Supporting Information). Specifically, the g value (361 nm) of 5 s UV‐treated samples had an obvious increase after the UV‐induced disassembly‐reassembly cycle (Figure 3c; Figure S21, Supporting Information sample without UV treatment (labeled as 0s): 0.052; 5 s UV treatment: 0.088), while the g values of 20 and 60 s treated samples decreased to 0.032 and 0.021, respectively, suggesting the amplification of optical asymmetry after 5 s UV exposure. By time‐dependent CD spectra, we observed that the 20, 60 s UV treated samples needed more time to accomplish reconstruction (Figure 3d) compared to that of 5 s treated ones, further implying the UV irradiation time‐dependent reconstruction process.

### 2.2 Reversible Electron Transfer Between LPF and NDIAPY

Thus, understanding the role of UV irradiation is the key to unraveling the mechanism of amplification of scale as well as the g value of helixes. UV spectra indicated that LPF‐NDIAPY co‐assemblies show a characteristic absorbance peak at 382 nm (Figure 3e), however, this peak decreased and a new peak at 450 nm appeared after 5 s UV irradiation (Figure 3e). It indicated the partial formation of NDIAPY radical anions induced by electron transfer (ET) from LPF to NDIAPY.^[^ ^63^, ^64^ ^]^ Meanwhile, 20 s UV irradiation resulted in a further decrease of the peak at 382 nm, and it disappeared after 60 s UV treatment, suggesting the total electron transfer. These results therefore indicated that the amplification of g value and scale of helixes after 5 s UV irradiation should be dependent on the certain incomplete ET. Moreover, the ratio of electron transfer in 5 s/20 s UV‐treated samples was obtained by linearly fitting the 5 s/20 s UV‐treated spectra via LPF‐NDIAPY without electron transfer (0 s UV‐treated sample) and LPF‐NDIAPY with total electron transfer (60 s UV‐treated sample) (Figure S23, Supporting Information). We observed that 5 s UV‐treated spectra = 9.5% 0 s UV‐treated spectra + 90.5% 60 s UV‐treated spectra, and 20 s UV‐treated spectra = 0.5% 0 s UV‐treated spectra + 99.5% 60 s UV‐treated spectra. It therefore indicated that the ratio of electron transfer in 5 s/20 s UV‐treated samples was ≈90.5%, and ≈99.5%, respectively. Furthermore, by monitoring the UV peak at 450 nm (Figure 3f), the intensity decreased after removing UV light, and totally disappeared within ≈50 s for 5 s treated sample, 170 s for 20 s treated sample, and 260 s for 60 s UV treated sample, indicating the complete recovery of NDIAPY radical anions to monomers.

Density functional theory (DFT) was employed to further study the photo‐induced ET process. The simulated UV peaks of NDIAPY single‐electron radical anion and its monomer were obtained from time‐dependent density functional theory (TDDFT) calculations, which were located at 480 and 370 nm (Figure 3h; Figure S22, Supporting Information), respectively. These results were consistent with experimental UV spectra of the NDIAPY radical anion and its monomer, supporting the formation of the NDIAPY radical anion by UV irradiation. To elucidate how the ET process, the excited‐state structures were obtained by TDDFT to study which packing modes favored the ET process. The dimer of LPF and NDIAPY was first optimized based on the hydrogen bonds between the carboxylic group of LPF and the pyridine of NDIAPY. However, the electron of LPF could not transfer to NDIAPY (Figure 3i; Figures S24 and S25, Supporting Information). Furthermore, the packing mode based on the close contact between the phenyl core of LPF and the NDI core of NDIAPY was simulated. Net 0.36 e electron successfully transferred to NDIAPY after exciting this optimized dimer (Figure 3j; Figures S26,S27 and S30, Supporting Information). Interestingly, UV spectra of Bocphe‐NDIAPY mixture revealed ET from phenylalanine (Phe) group of Bocphe to NDIAPY (Figure 3k, after 5 s or 20 s UV irradiation of Bocphe‐NDIAPY, a new UV peak at ≈470 nm was immediately detected, indicating that the phenylalanine (Phe) group could also interact with NDIAPY and transfer the electric electron). The dimer based on the interaction between Phe groups of LPF and NDI cores of NDIAPY was therefore optimized (Figure 3l; Figures S28–S30, Supporting Information), in which net 0.66 e electron of LPF transferred to NDIAPY an excited state. These results indicated that the ET between LPF and NDIAPY could be realized in two manners: one is from the phenyl core to the NDI core; and another is from the Phe group to the NDI core.

### 2.3 Electron Transfer Assisted Secondary Nucleation for Chirality Evolution

**Figure 4:**
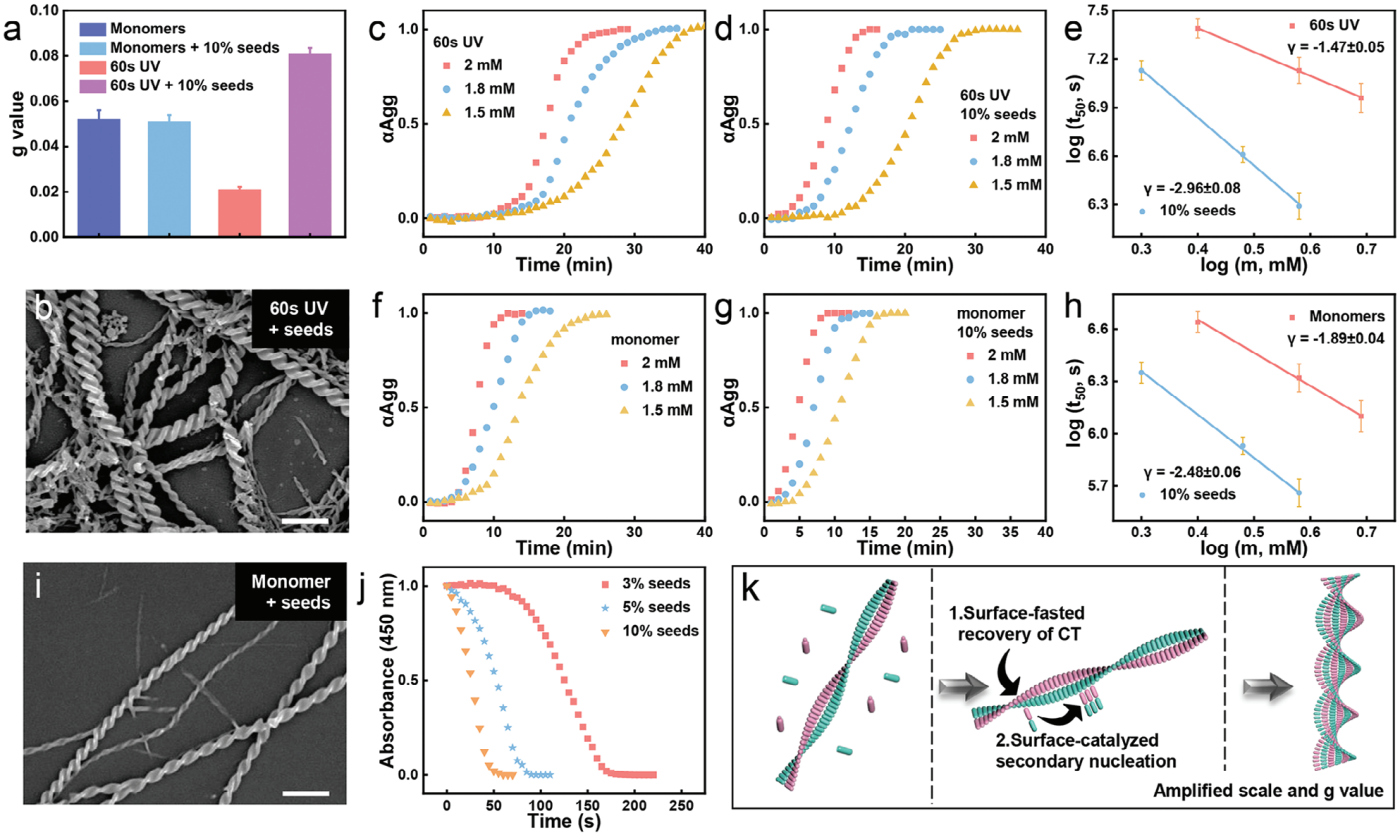
a) the g value of monomer systems with or without 10% seeds and 60 s UV treated samples with or without 10% seeds. (b) SEM images of 60 s UV‐treated samples with 10% seeds. Scale bar: 1 µm. c,d,f,g) Time‐dependent αAgg by monitoring the CD signal at 361 nm. (c) 60 s UV treated samples, (d) 60 s UV treated samples with 10% seeds, (f) monomer systems, (g) monomer systems with 10% seeds. e,h) Double logarithm plots of t_50_ with the monomer concentration for unseeded and seeded growth. (e) 60 s UV treated samples, (h) monomer systems. i) SEM images of the monomer system with 10% seeds. Scale bar: 1 µm. j) Time‐dependent normalized absorbance at 450 nm for 60 s UV‐treated samples with 3%, 7%, and 10% seeds. k) Schematic illustration of scale amplification by surface accelerated recovery of ET and surface catalyzed secondary nucleation.

However, it was still unclear for the necessary role of incomplete ET in amplifying scale and g value. Detailed kinetic analysis of the evolution process was studied by time‐dependent CD spectra. For 60 s UV‐treated LPF‐NDIAPY co‐assembly (2 mM) (Figure S31, Supporting Information), the CD peaks at 361 nm showed a non‐linear kinetic profile with time, indicating a cooperative growth mechanism. The half‐time (t_50_), defined as the time required for 50% completion of the growth process, was 1020 s. By immediately adding LPF‐NDIAPY co‐assembly with different ratios (3%, 5%, 10%) into the systems after 60 s UV irradiation, the reconstruction process of LPF‐NDIAPY (2 mM) significantly accelerated as evidenced by the decrease in t_50_ (930 s for 3%, 810 s for 5%, 540 s for 10%). It suggested that the undestroyed LPF‐NDIAPY co‐assembly should play as a seeds to trigger the chirality evolution process.^[^ ^62^ ^]^ Moreover, the increased g value (**Figure** **4**a; Figure S32, Supporting Information) and amplification of scale of helixes (Figure 4b; Figure S33, Supporting Information) were only observed in 10% LPF‐NDIAPY seeds added samples, therefore indicating that this chirality evolution was dependent on the ratio of undestroyed LPF‐NDIAPY co‐assemblies, which echoed the results of UV spectra.

To further explore the mechanism of both scale and g value amplification, in‐depth kinetic analyses of the seeded growth were conducted, an increase in width and g value in the presence of seeds is suggestive of a seeded growth pathway such as surface‐catalyzed secondary nucleation, which can initiate growth along the width direction. To date, the models to distinguish the primary and secondary nucleation events have been well established. For example, the half‐time (*t_50_*) of the growth process is related to the initial monomer concentration (*m*) via a rate law equation as follows in Equation 1:

(1) $$\log\left(t_{50}\right)=\gamma \;\log\left(m\right)+\mathrm{constant}$$

where γ is the scaling exponent and is related to the reaction order (*n_2_*) of the dominant self‐assembly mechanism. The scaling exponent for the secondary nucleation‐dominated self‐assembly process is described as Equaiton 2:

(2) $$\gamma =-\left(n_{2\;}+1\right)/2$$

thus, the γ extracted from a double logarithm plot of *t_50_* with *m* can shed light on the nature of the nucleation process for the LPF‐NDIAPY system. Concentration‐dependent measurements were then performed. For the 60 s UV treated samples without the addition of seed, with increasing LPF‐NDIAPY concentration from 1.5 to 2 mM, resultant growth kinetics exhibited a decrease in the half‐time (t_50_) (1620, 1260, 1020 s for 1.5, 1.8, 2 mM, respectively) (Figure 4c). The double logarithmic plot of *t_50_* with LPF‐NDIAPY concentration (*m*) showed a slope of −1.47 (*γ*) with a reaction order (*n_2_*) of 2 (Figure 4e, red line), implying the existence of secondary nucleation events. However, in the presence of 10% seeds, the decrease in t_50_ from 1250 s for 1.5 mM to 540 s for 2 mM was observed (Figure 4d). The scaling exponent (*γ*) showed a remarkable decrease from −1.47 to −2.96 (*n_2_* = 5) (Figure 4e, blue line), suggesting the greater propensity toward surface‐catalyzed secondary nucleation elongation during the seed‐induced reconstruction process. Above all, it was the surface‐catalyzed secondary nucleation that triggered lateral growth of co‐assembly, subsequently resulting in width and g value increase.

Meanwhile, the width (Figure 4i) as well as g value (Figure 4d; Figures S34 and S35, Supporting Information) in the seeded monomer system were all consistent with those of the unseeded sample, indicating that the amplification of scale and g value could not be realized by adding seeds into the monomer solution of LPF and NDIAPY. Besides, the final UV spectra of these systems show accordant absorption at the same concentration (Figure S34, Supporting Information), indicating the consistent extent of assembly between this monomeric system and UV‐treated systems. According to the kinetic analyses, the half‐time (*t_50_*) of unseeded monomer systems increased from 450 to 780 s with a decrease of the concentration from 2 to 1.5 mM (Figure 4f). Meanwhile, the half‐time (*t_50_*) also decreased in the presence of 10% seeds (Figure 4g, 580, 420, 290 s for 1.5, 1.8, and 2 mM, respectively). The scaling exponent (*γ*) showed a decrease from −1.89 for unseeded systems to −2.48 for seeded samples (Figure 4h). It indicated the occurrence of secondary nucleation events in seeded monomer systems. However, the above scaling exponent (*γ* = −2.48) for seeded monomer systems was higher than that for the 60 s seeded UV‐treated‐samples (*γ* = −2.96), indicating the lower tendency toward surface‐catalyzed secondary nucleation elongation in seeded monomer‐systems. Thus, it highlighted the irreplaceable role of the UV‐induced ET process in realizing both width and g value amplification of LPF‐NDIAPY helices.

To further unravel the mechanism of chirality amplification, the influence of seeds on the recovery process of NDIAPY radical anions back to monomers was studied by UV spectra (Figure 4j). Surprisingly, upon increasing the concentration of seeds from 3% to 10%, the recovery time had a significant suppression from ≈170 to ≈45 s. Thus, the seeds can accelerate the recovery of ET (surface‐accelerated recovery of ET) (Figure 4k), which might facilitate the propensity toward surface‐catalyzed secondary nucleation elongation in UV‐treated samples compared to monomer‐system. It indicated the fact that ET assisted secondary nucleation.

**Figure 5:**
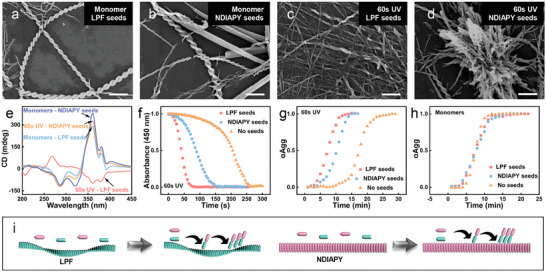
a–d) SEM images of (a) 60 s UV‐treated samples with LPF seeds, (b) monomer system with LPF seeds, (c) 60 s UV‐treated samples with NDIAPY seeds, and (d) monomer system with NDIAPY seeds. Scale bar for a–d: 1 µm. e) CD spectra of 60 s UV‐treated samples with LPF seeds, monomer system with LPF seeds, 60 s UV‐treated samples with NDIAPY seeds, and monomer system with NDIAPY seeds. f) Time‐dependent UV peak at 382 nm for 60 s UV treated sample, 60 s UV treated sample with LPF seeds, and 60 s UV treated sample with NDIAPY seeds. g) Time‐dependent CD peak at 361 nm for 60 s UV treated sample, 60 s UV treated sample with LPF seeds, and 60 s UV treated sample with NDIAPY seeds. h) Time‐dependent CD peak at 361 nm for monomeric sample, monomeric sample with LPF seeds, and monomeric sample with NDIAPY seeds. i) Schematic illustration of the secondary nucleation for 60 s UV treated sample with LPF seeds and 60 s UV treated sample with NDIAPY seeds.

Moreover, the ET assisted secondary nucleation was also studied using LPF or NDIAPY assemblies as seeds. LPF seeds presented right‐handed helical nanofibers, while nanobelts for NDIAPY seeds (Figure S6, Supporting Information). After adding 100% seeds of LPF or NDIAPY into the monomer system, the co‐assembled structures of LPF‐NDIAPY with ≈0.052 g value were still observed (**Figure** **5**a,b,e; Figure S37, Supporting Information), indicating the negligible effect of LPF/NDIAPY seeds on the co‐assembly process of LPF and NDIAPY monomers. However, adding 100% LPF seeds into a 60 s UV treated‐system gave rise to the formation of right‐handed nanofibers (Figure 5c) which presented opposite handedness compared to the co‐assembled left‐handed helices of LPF‐NDIAPY, and the corresponding CD spectra showed a positive peak at 268 nm and a negative peak at 361 nm (Figure 5e). Brush‐like helical fibers (Figure 5d) were formed in UV treated systems with 100% NDIAPY seeds. UV spectra indicated that the recovery of ET was also accelerated by the seeds of LPF/NDIAPY (Figure 5f). Besides, the half‐time (*t_50_*) of the 60 s UV treated‐system was decreased in the presence of seeds of LPF/NDIAPY (Figure 5g), while half‐time for monomer systems with or without LPF/NDIAPY seeds did not display an obvious difference (Figure 5h). It indicated that the LPF/NDIAPY seeds‐accelerated recovery of ET was the prerequisite for subsequent secondary nucleation−elongation events. Besides, it also implied the different chirality evolutions were triggered by the UV treated‐systems with LPF (chirality inversion) or NDIAPY (morphological transformation) seeds. Above all, the concept of ET assisted secondary nucleation−elongation for chirality evolution of self‐assembled nanohelices was built.

Finally, XRD experiments were performed to investigate the mechanism of chirality evolution triggered by electron transfer‐assisted secondary nucleation (Figure S38, Supporting Information). XRD patterns of LPF, NDIAPY, LPF‐NDIAPY, 5 s/60 s UV‐treated LPF‐NDIAPY samples were obtained by a slow scan rate of 0.5°/min. We first found out the existence of LPF and NDIAPY characteristic XRD peaks in the LPF‐NDIAPY sample, indicating that some LPF and NDIAPY self‐assembled structures were also formed, which is known as self‐sorting. However, the characteristic XRD peaks of LPF and NDIAPY vanished in the 5 s UV‐treated LPF‐NDIAPY sample, indicating that the secondary nucleation process could significantly enhance the co‐assembly tendency of LPF and NDIAPY. Thus, asymmetry amplification is ascribed to the formation of more LPF‐NDIAPY assemblies. Besides, the XRD pattern of the 60 s UV‐treated LPF‐NDIAPY sample showed weak diffraction peaks with broader full width at half maximum compared to that of the 5 s treated sample. This observation suggested that the order degree of co‐assembly has decreased with increasing UV irradiation time, which may account for the decrease in asymmetry for the 60 s UV‐treated LPF‐NDIAPY samples. Besides, this observation also indicated that the primary nucleation triggered co‐assembly process is obviously disturbed by the UV irradiation, which resulted in the increased of possibility secondary nucleation events and further gave rise to the ordered co‐assembly of the UV‐treated LPF‐NDIAPY system. Moreover, this decreased tendency toward primary nucleation after UV irradiation might also increase the chance of secondary nucleation events at other seeds such as LPF, leading to the chirality of the system being regulated by LPF seeds.

## 3 Conclusion

In summary, the chirality evolution of supramolecular helices was achieved via ET assisted secondary nucleation. The ET from LPF to NDIAPY in the co‐assemblies can be triggered by UV light, leading to the formation of NDIAPY radical anions and the partial disassembly of the helices. Then, the removal of UV light resulted in the spontaneous reversion of radical anions into monomers, and the surface of residual co‐assemblies can accelerate the reversion process. Furthermore, surface accelerated reversion of ET facilitates the secondary nucleation‐elongation events, giving rise to the formation of scale‐amplified and g vale‐increased left‐handed helices. Meanwhile, chirality evolution controlled by ET assisted secondary nucleation process can be also realized by adding the prepared LPF‐NDIAPY co‐assemblies into the total ET system. These results illustrate the feasibility of chirality evolution induced by secondary nucleation in an artificial system, which may provide a universal methodology to regulate other chirality evolution and accelerate the precise synthesis of chiral materials through rational control of the nucleation process.

## Conflict of Interest

All authors declare no competing interests.

## Supporting information



Supporting Information

## Data Availability

The data that support the findings of this study are available from the corresponding author upon reasonable request.
