# Supplementary material for: Chirality Evolution of Supramolecular Helices by Electron Transfer Assisted Secondary Nucleation
Source: Adv Sci (Weinh). 2024 Dec 16;12(5):2408499. doi: 10.1002/advs.202408499 (PMC11792056; doi:10.1002/advs.202408499)
Supplement: Supplementary file 1 — Supporting Information [file ADVS-12-2408499-s001.docx]

Supporting Information

Chirality Evolution of Supramolecular Helices by Charge Transfer Assisted Secondary Nucleation

Laiben Gao^a^, Xiaoqiu Dou*^a^, Chao Xing^a^, Kaikai Yang^a^, Changli Zhao^a^, and Chuanliang Feng*^ab^

^a^State Key Lab of Metal Matrix Composites, School of Materials Science and Engineering, Shanghai Jiao Tong University, Shanghai, 200230 (P.R. China).

^b^Shanghai Key Laboratory for Molecular Engineering of Chiral Drugs, Shanghai Jiao Tong University, Shanghai, 200230 (P.R. China)

*Corresponding author

E-mail: clfeng@sjtu.edu.cn and douxiaoqiu@sjtu.edu.cn.

1. Experimental Section

**1.1 Materials**: L-phenylalanine methyl ester hydrochloride, 1,4-benzenedicarbonyl dichloride, 1,4,5,8-Naphthalenetetracarboxylic Dianhydride, 4-Pyridinemethaneamine, triethylamine (Et_3_N), Dichloromethane (DCM), Methanol (CH_3_OH), Sodium hydroxide (NaOH), N, N-Dimethylformamide (DMF), hydrochloric acid (HCl) and were purchased from Aladdin Chemicals.

**1.2 Characterization:** ^1^H Nuclear Magnetic Resonance (^1^H NMR) spectroscopy was performed on a Bruker Advance III 400 Instrument operating at 400 MHz. DMSO-*d_6_* was used as the solvent for ^1^H NMR measurements. DOSY spectra were recorded on Bruker Advance III 600 Instrument operating at 600 MHz. The mixture of HFIP-*d_2_* and D_2_O (1:9, v/v) was used as solvent (Concentrations: for LPF, LPF: 2 mmol/L; LPF-NDIAPY, LPF: 2 mmol/L, NDIAPY: 2 mmol/L.). Electrospray Ionization Mass Spectrometry (LC-MS) was recorded on a Bruker impact II Instrument. Methanol was used as the solvent for LC-MS measurements. CD spectra were recorded on a JASCO J-1500 CD spectrometer. UV-vis spectroscopy was recorded on an Evolution 201. For CD and UV experiments, all scans were performed at a scan speed of 500 nm/min with a data pitch of 0.5 nm at the room temperature. Concentrations: for LPF, LPF: 2 mmol/L; LPF-NDIAPY, LPF: 2 mmol/L, NDIAPY: 2 mmol/L. SEM images were obtained using a FEI QUANTA 250 Microscope. The samples were prepared by depositing solutions of assemblies on silicon wafers. After drying under vacuum, the samples were coated with a thin layer of Au. FTIR spectra were taken using with a ThermoFisher Scientific Nicolet iS5 instrument.

1.3 **Synthesis**

**Synthesis of LPF**. 1,4-benzenedicarbonyl dichloride (2.6 g, 13.0 mmol) in dichloromethane (DCM, 30 ml) was added dropwise to a solution triethylamine (Et_3_N, 10.0 ml, 72.9 mmol) of and L/D-phenylalanine methyl ester hydrochloride (6.0 g, 26.1 mmol) in dry DCM (150 ml). The solution was stirred at room temperature for 24 h. The solvents were evaporated and the residue was subsequently dissolved in ethanol (100 ml). After filtration, the undissolved substance was collected and dried to give the dimethyl ester of L/DPF (4.76 g, 9.8 mmol, 75%). For the hydrolysis, aqueous NaOH (10 ml, 2.0 M) was added to a cooled (25 °C) suspension of the dimethyl ester of L/DPF (4.76 g, 9.8 mmol) in MeOH (50 ml). The mixture was slowly brought back to room temperature and stirred for 24 hours and a clear solution was obtained. The solution was then acidified with 3.0 M HCl until pH value no more than 3.0 and gel-like precipitate formed. The gel phase was filtered, washed with deionized water, and finally dried in the vacuum oven to give L/DPF (3.96 g, 8.6 mmol, 87%). Overall yield: 66%.^1^H NMR (500 MHz, DMSO-d6) δ 12.82 (s, 1H), 8.83 (d, J = 8.1 Hz, 1H), 7.84 (d, J = 3.3 Hz, 2H), 7.35 – 7.23 (m, 4H), 7.22 – 7.14 (m, 1H), 4.62 (ddd, J = 10.7, 8.1, 4.5 Hz, 1H), 3.20 (dd, J = 13.8, 4.5 Hz, 1H), 3.07 (dd, J = 13.9, 10.7 Hz, 1H), 2.52 (d, J = 1.8 Hz, 18H). LC-MS (m/z) for C_26_H_24_N_2_O_6_ calcd.460.1689; found 461.1699 [M+H]^+^.

**Synthesis of NDIAPY.** 1,4,5,8-Naphthalenetetracarboxylic Dianhydride (2.68 g, 10.0 mmol) and 4-Pyridinemethaneamine (2.36 g, 22.0 mmol) were refluxed under 140 ^o^C in DMF (50 ml) under N_2_ for 12 h. The solid was isolated by vacuum filtration and washed by acetonitrile and acetone. Yield: 2.82 g (6.3 mmol, 63%). ^1^H NMR (500 MHz, DMSO-*d_6_*) δ 8.74 (s, 4H), 8.52 – 8.49 (m, 4H), 7.42 – 7.40 (m, 4H), 5.31 (s, 4H). LC-MS (m/z) for C_26_H_16_N_4_O_4_ calcd.448.12; found 449.1241[M+H]^+^.

**1.4 DFT simulation**

All calculations were carried out with the Gaussian 16 software.^[1]^ Density functional theory (DFT) calculations were carried out with the M06-2X functional with the combination of the Grimme’s D3 version of dispersion correction.^[2]^ The basis set of 6-31G (d, p) were adopt for the geometry optimization and frequency calculations. The geometries were fully optimized without any structural constraints in the water solution with the SMD solvation model. The harmonic frequency calculations were carried out at the same level of theory to verify that all structures have no imaginary frequency.

**1.5 g value calculation**

g-factor was calculation by following formula:

g = CD(mdeg)/(32980 × Abs).

**1.6 kinetic experiments**

Firstly, LPF-NDIAPY seeds with concentration of 2, 1.8, 1.5 mM in HFIP/H_2_O (1:9, v/v) were prepared for using to seeded systems with corresponding concentration of 2, 1.8, 1.5 mM, respectively. For the LPF-NDIAPY monomeric system with seeds, we take the LPF-NDIAPY (2 mM) with 10% seeds as the example to illustrate the experimental process. 100 μl HFIP of LPF-NDAIPY is firstly added into 900 μl H_2_O, and 100 ul seeds of LPF-NDIAPY (2 mM) are immediately injected into above systems, giving the monomeric seeded system. For the UV treated LPF-NDIAPY with 10% seeds, UV-treated LPF-NDIAPY (2 mM) with 10% seeds is taken as the example. 60s UV irradiation is firstly performed on 1000 ul solution of LPF-NDIAPY assemblies (2 mM) in HFIP/H_2_O (1:9, v/v). Upon removal of UV light, 100 ul seeds of LPF-NDIAPY (2 mM) are immediately added into above systems, therefore giving the UV-treated seeded system.

2. Supplementary Figures


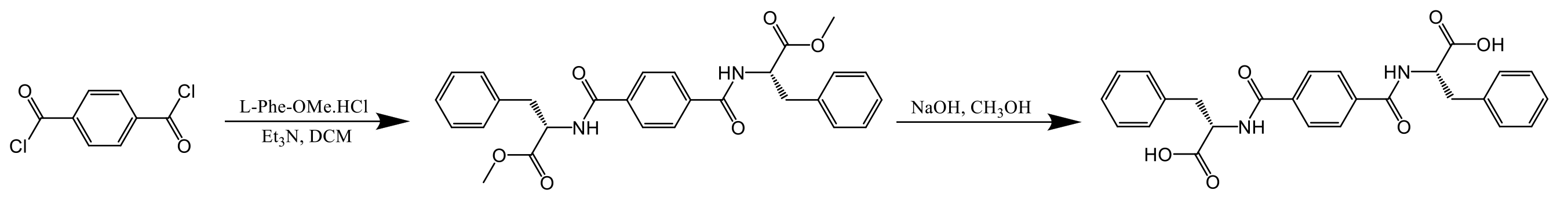


**Figure S1.** Synthetic route of LPF.


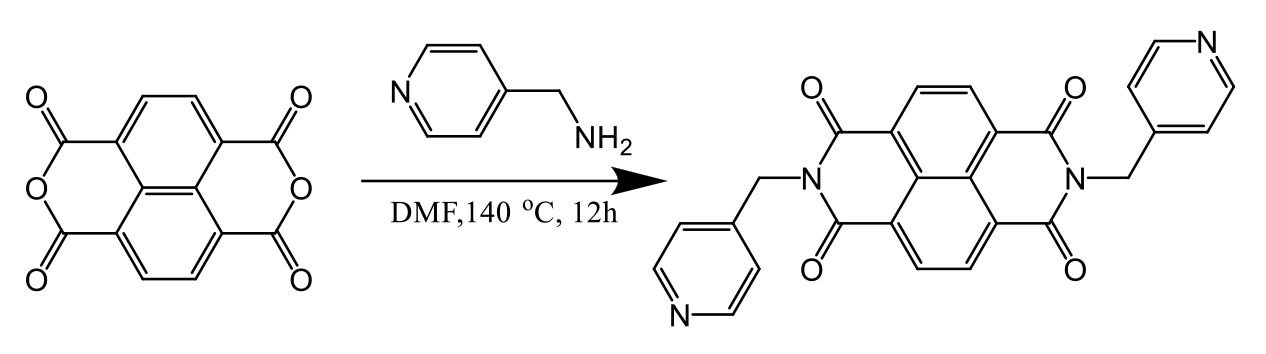


**Figure S2.** Synthetic route of NDIAPY.


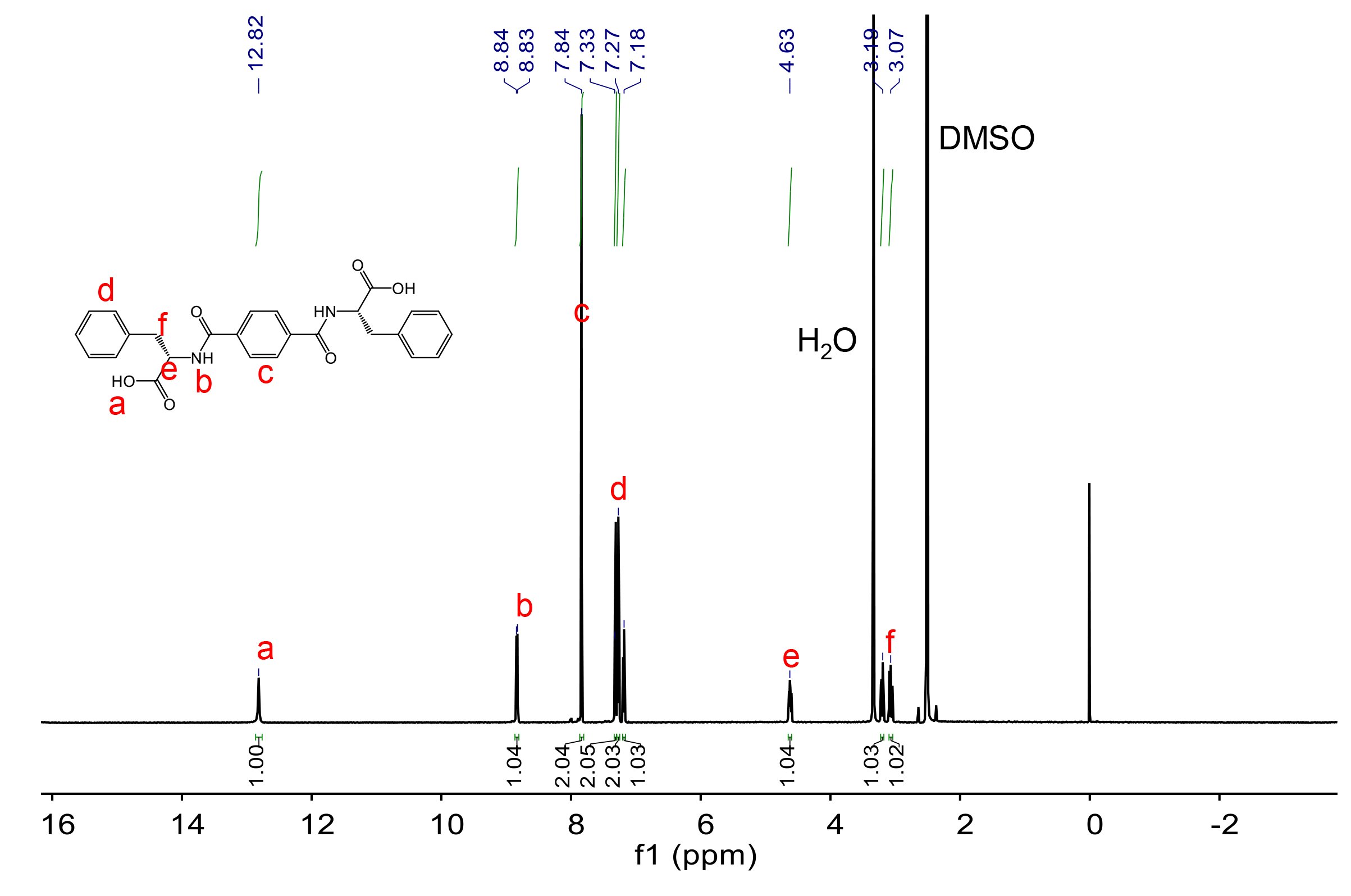


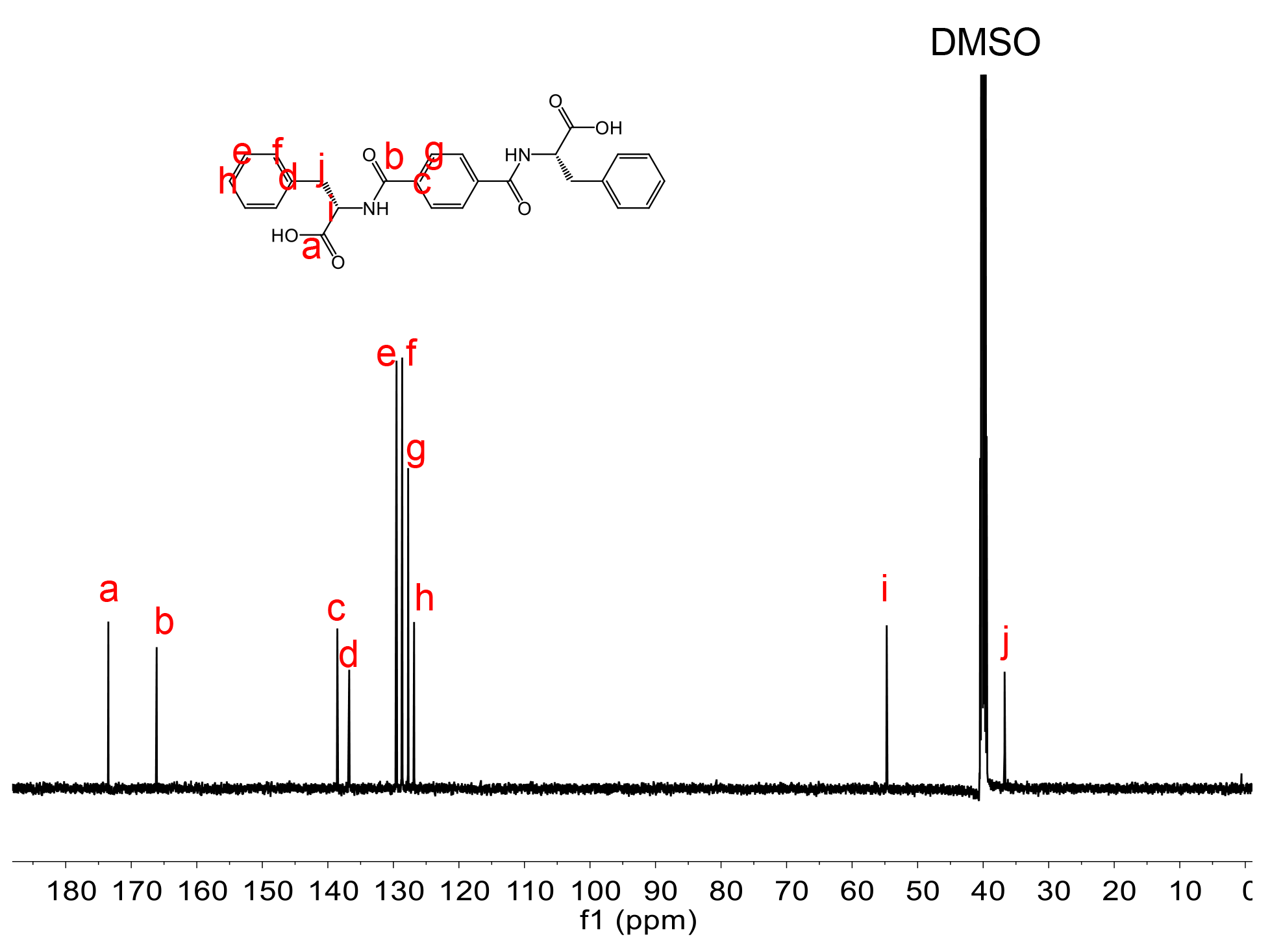


**Figure S3.** ^1^H NMR and ^13^C NMR spectra of LPF in DMSO-*d_6_*_._


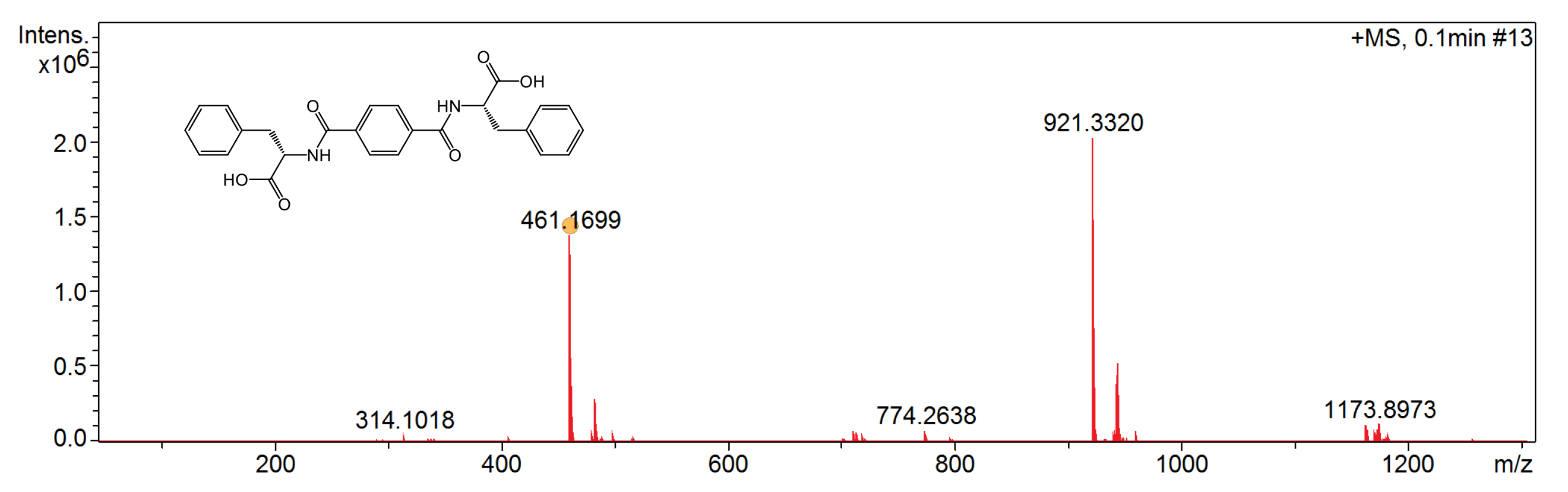


**Figure S4.** LC-MS spectrum of LPF in CH3OH. Type of ionization: Electron spray ionization (ESI). Positive mode. LC-MS (m/z) for C_26_H_24_N_2_O_6_ calcd.460.1689; found 461.1699 [M+H]+.


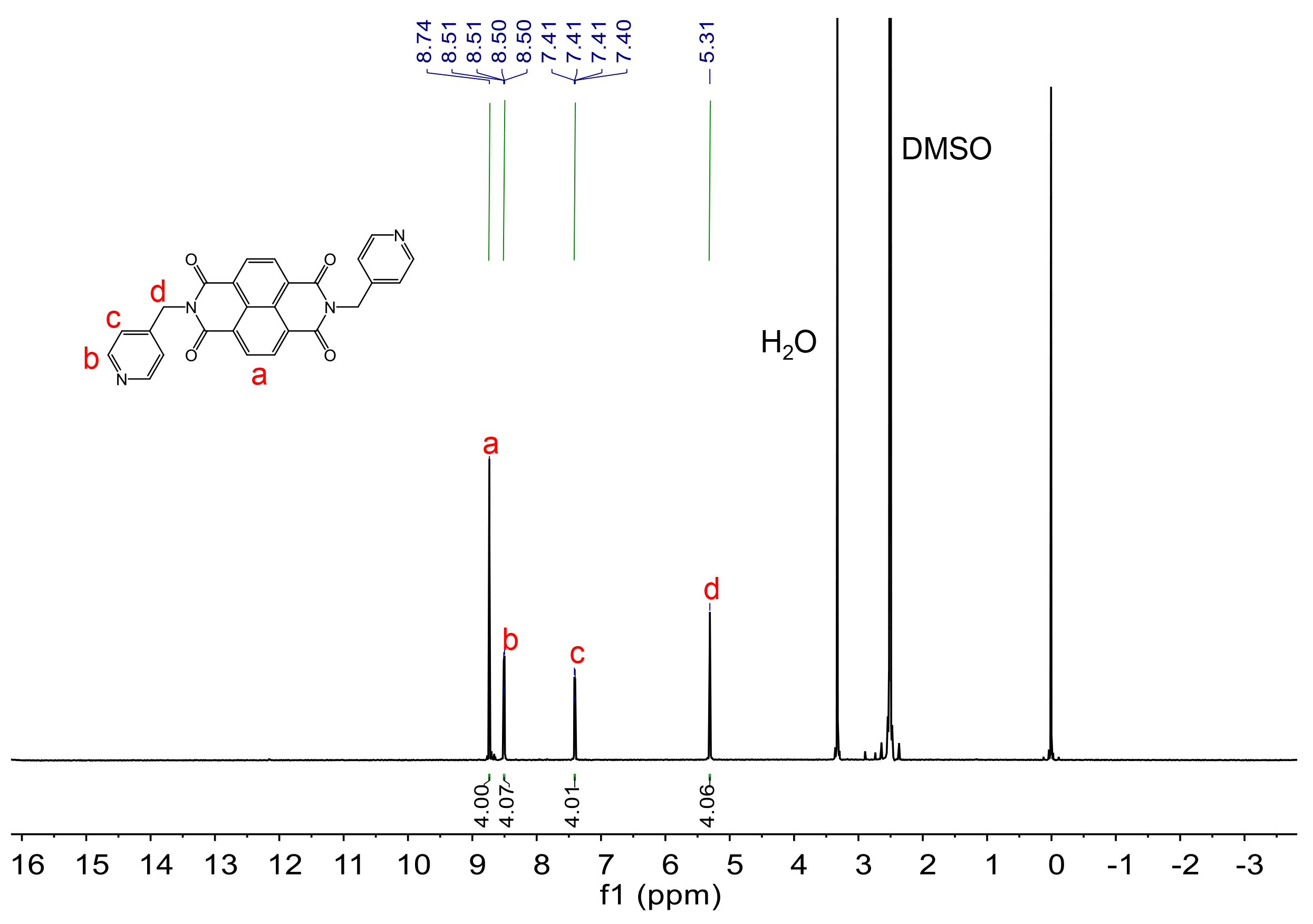


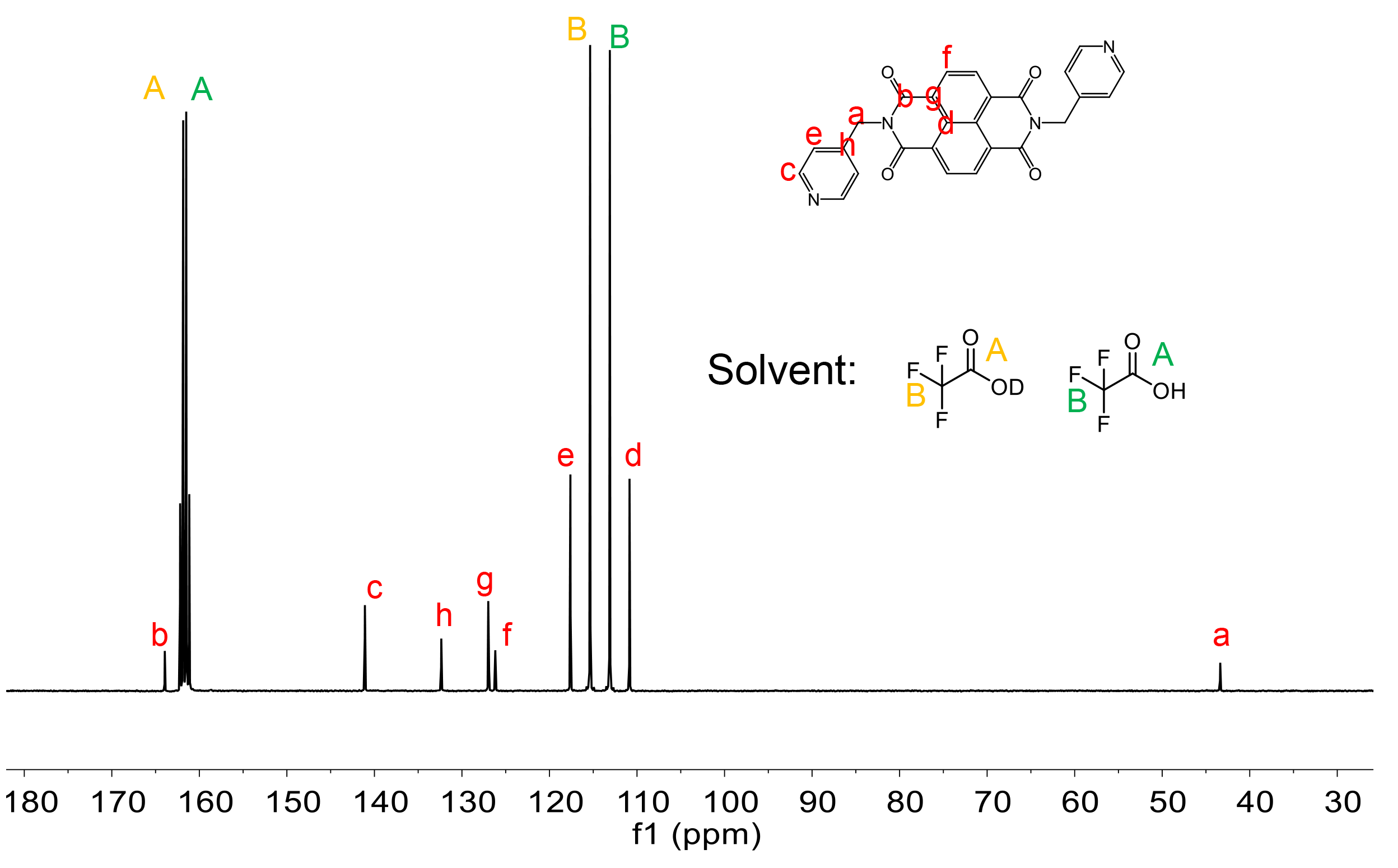


**Figure S5.** ^1^H NMR and ^13^C NMR spectra of NDIAPY in DMSO-*d_6_.*


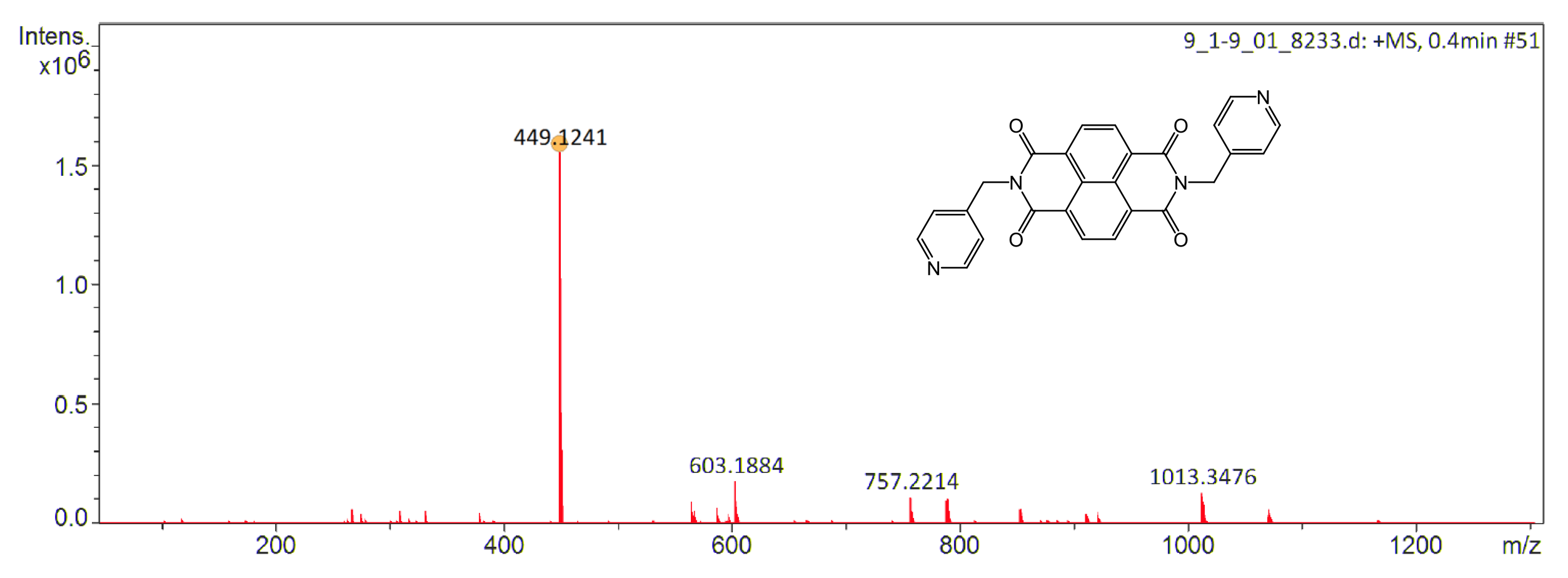


**Figure S6.** LC-MS spectrum of NDIPY in CH_3_OH. Type of ionization: Electron spray ionization (ESI). Positive mode.LC-MS (m/z) for C_26_H_16_N_4_O_4_ calcd.448.12; found 449.1241[M+H]+.


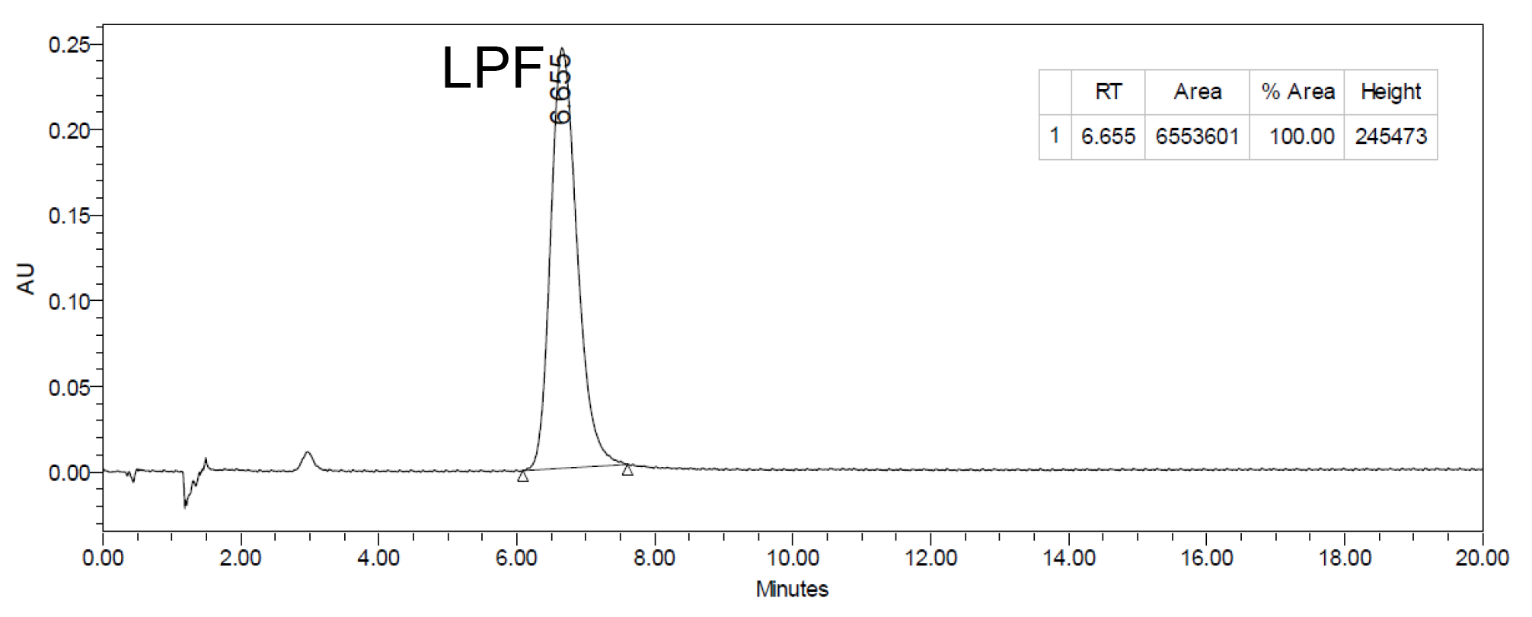


**Figure S7.** Chiral HPLC of LPF.


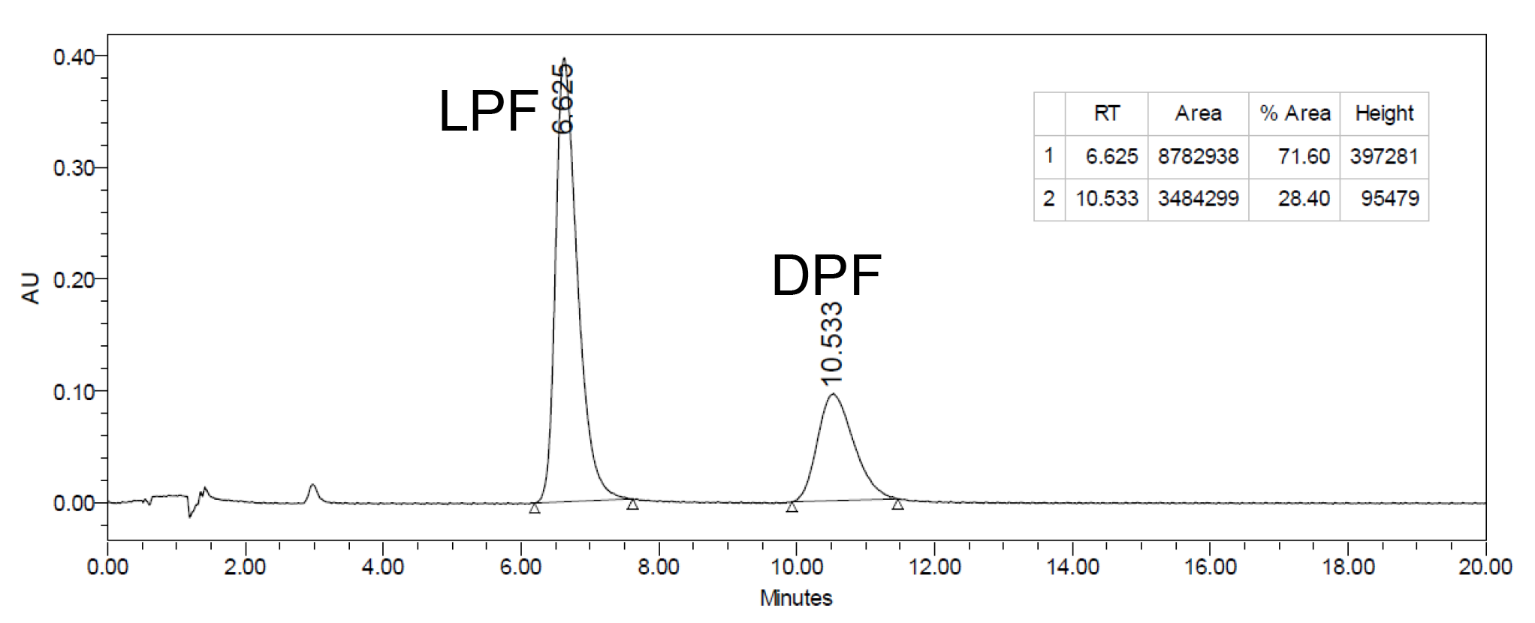


**Figure S8.** Chiral HPLC of the mixtures of LPF and DPF.


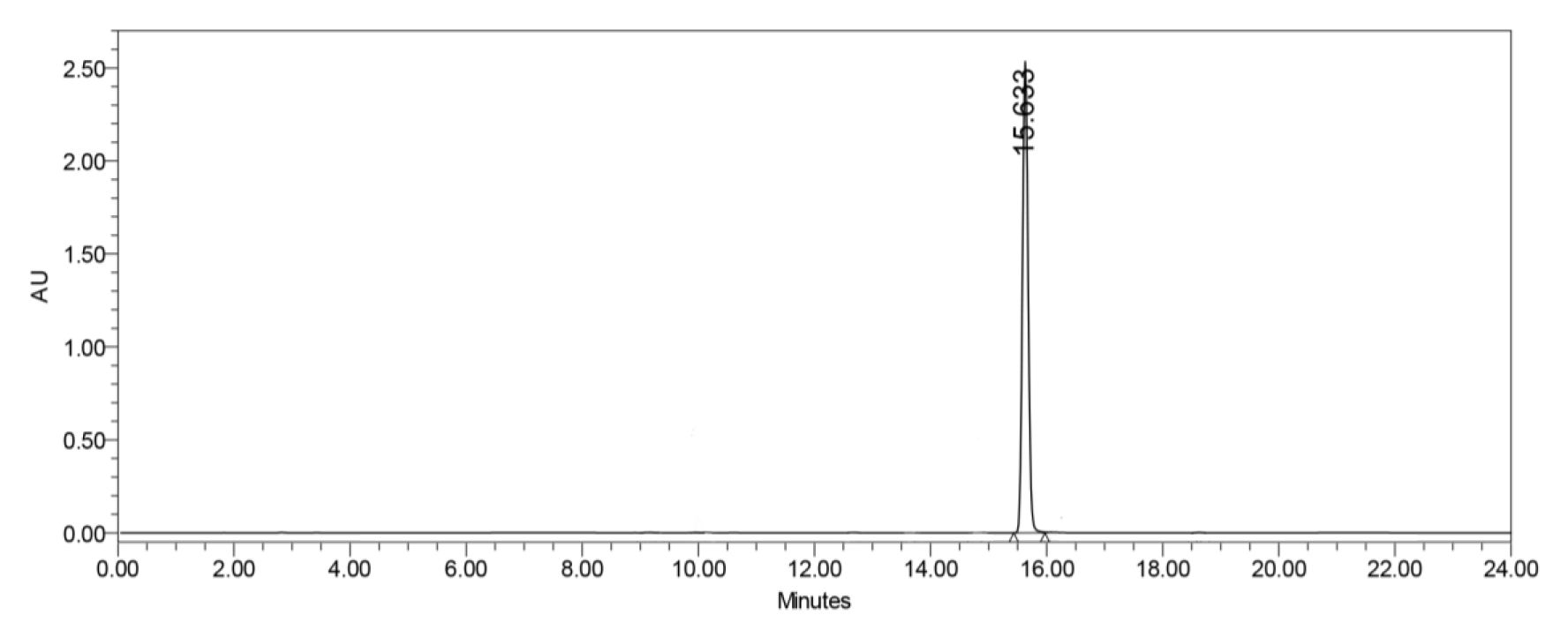


**Figure S9.** HPLC of NDIAPY.


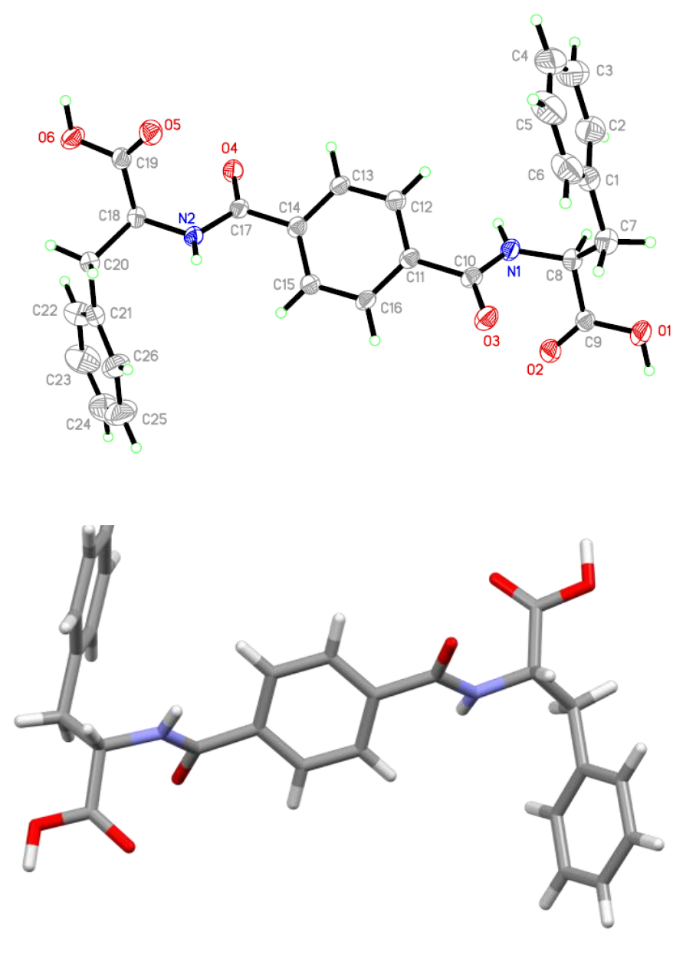


**Figure S10.** The single crystal of LPF. The crystal of LPF is cultured by slowly evaporating the hexafluoroisopropanol/p-xylene/water (3:7:0.1, v/v/v) solution of LPF at room temperature.


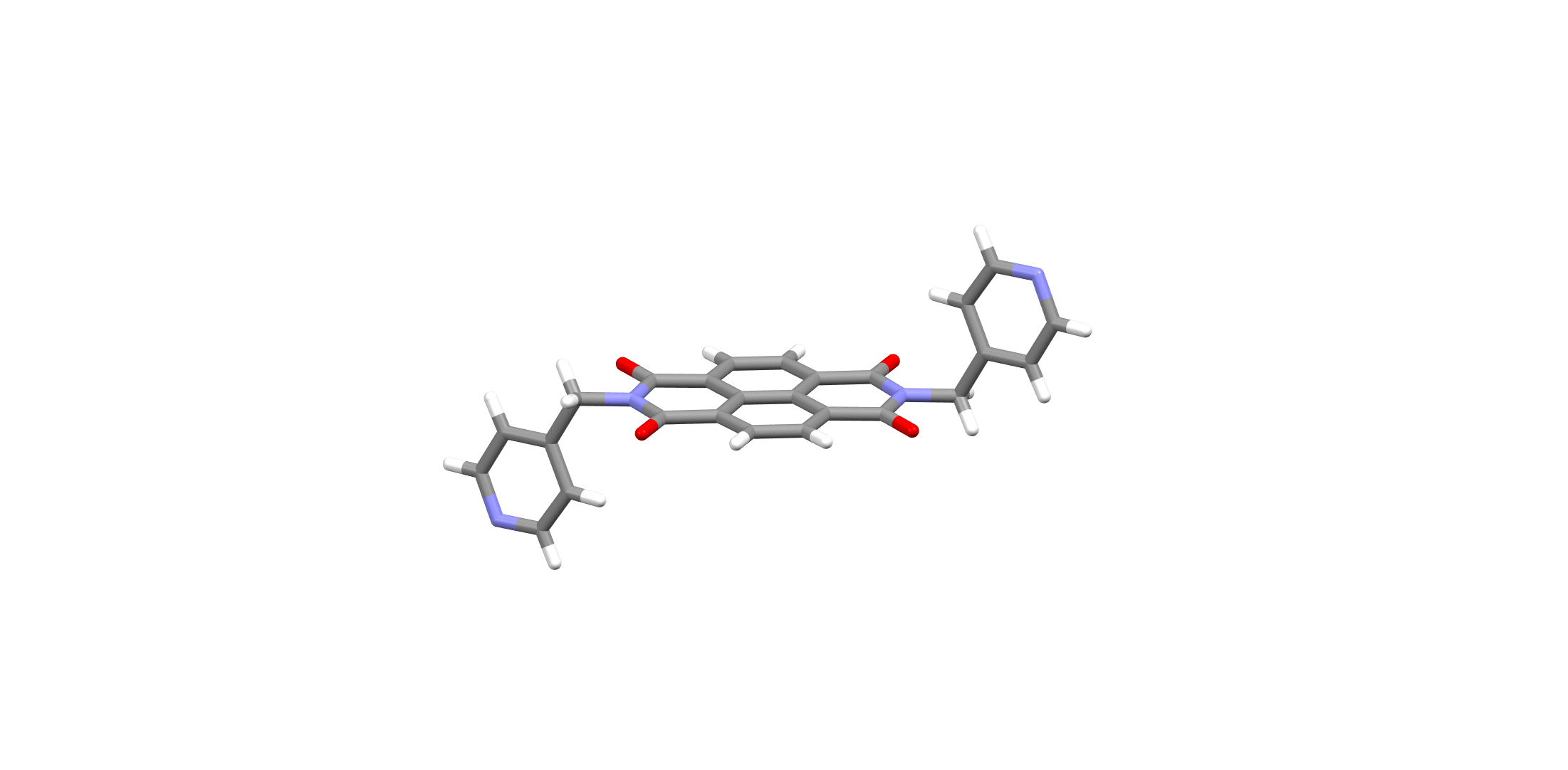


**Figure S11.** The single crystal of NDIAPY. The crystal of NDIAPY was obtained via solvent exchange between HFIP solution of NDIAPY and diethyl ether.


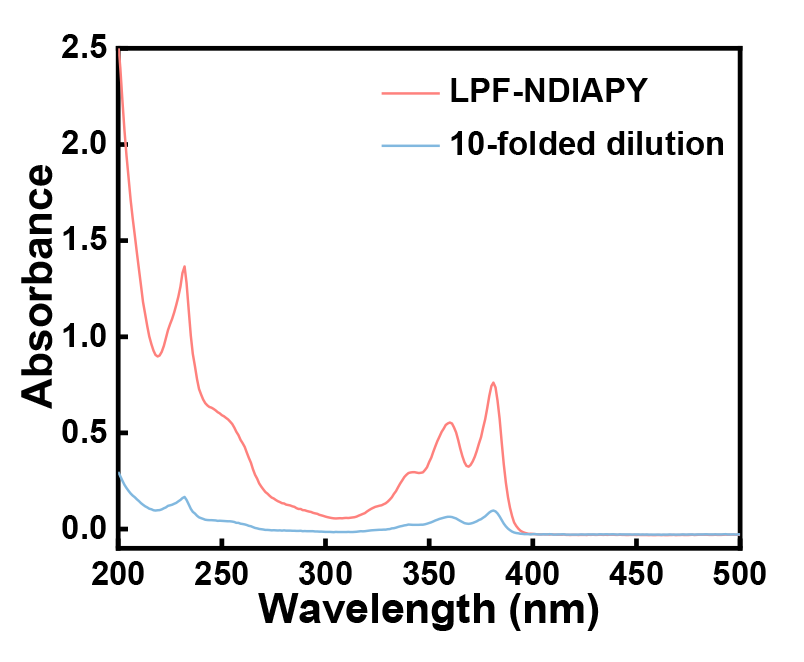


**Figure S12.** UV spectra of LPF-NDIAPY stock solution with or without 10-folded dilution.


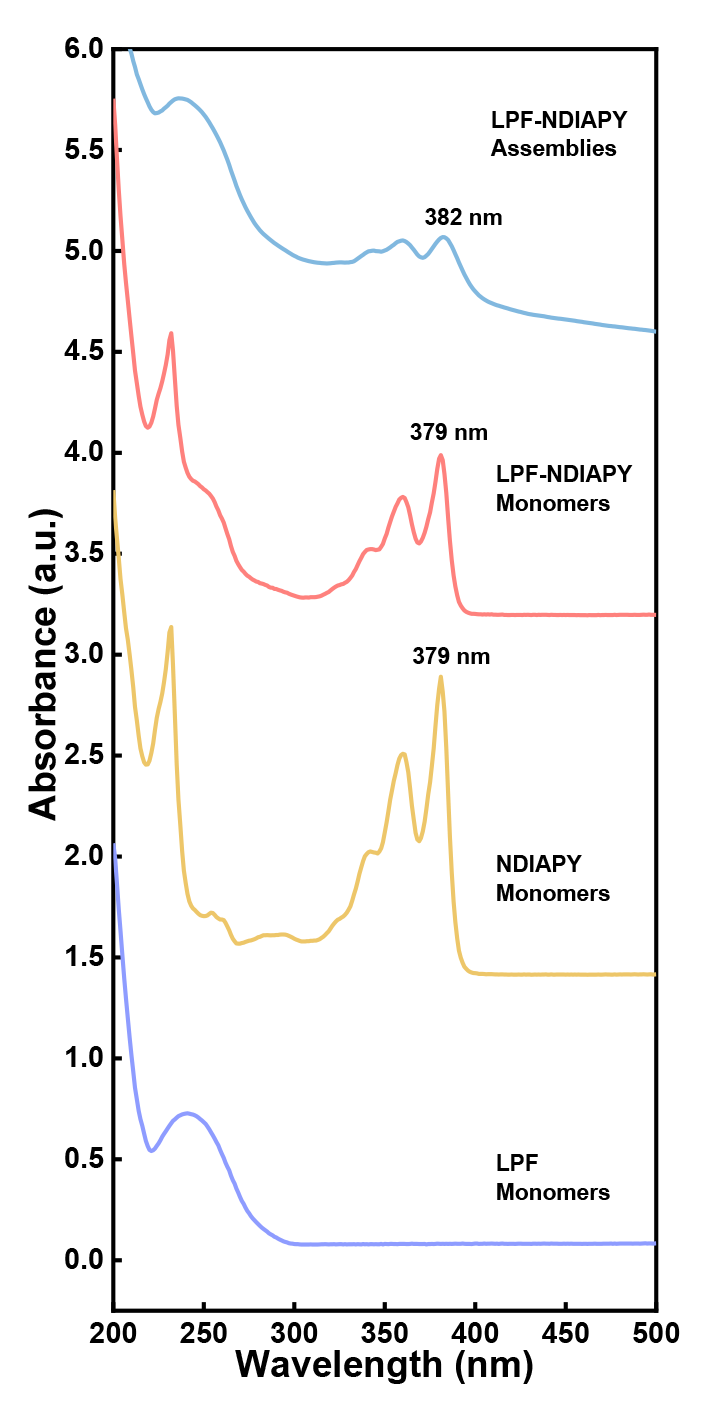


**Figure S13.** UV spectra of LPF-NDIAPY assemblies and monomers, NDIAPY monomers, LPF monomers.


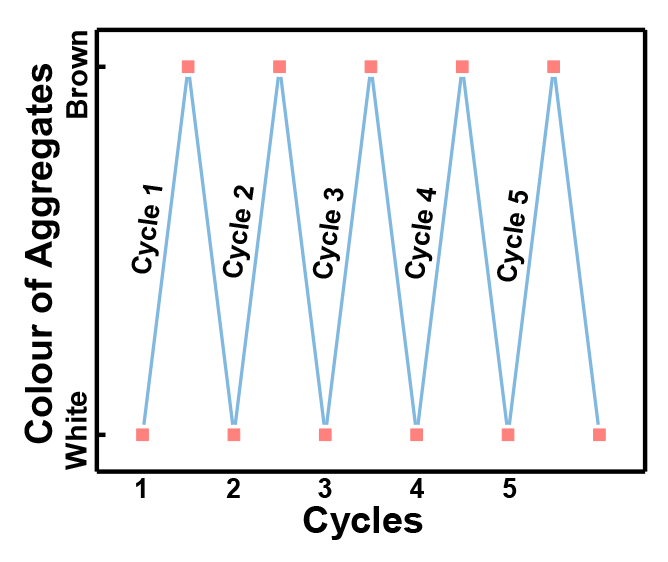


**Figure S14.** the reversible color switch by alternately opening and closing the UV irradiation for LPF-NDIAPY assemblies.


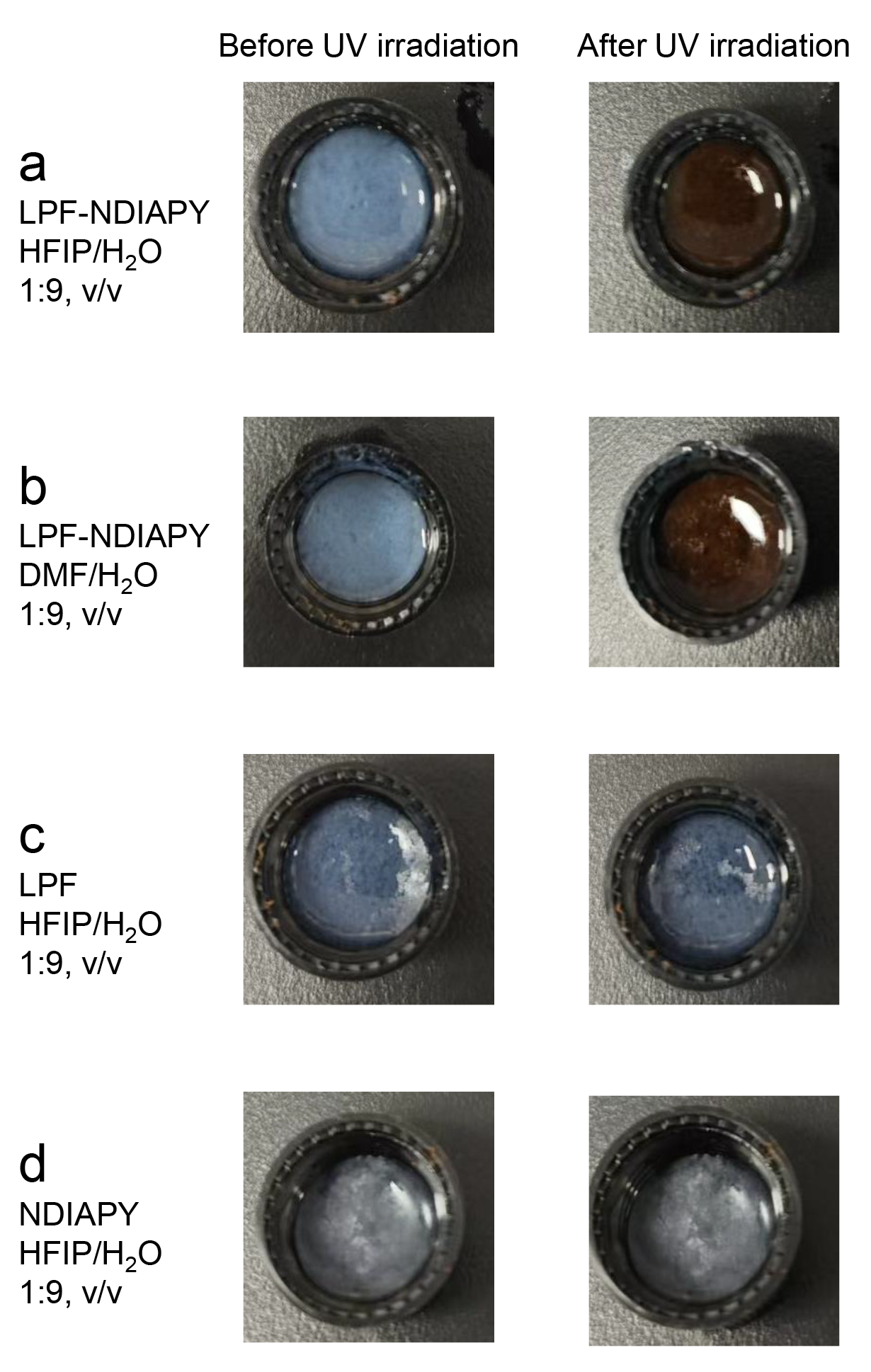


**Figure S15.** The macrophotograph before and after UV irradiation, (a), LPF-NDIAPY in HFIP/H_2_0 (1:9, v/v), (b), LPF-NDIAPY in DMF/H_2_0 (1:9, v/v), (C), LPF in HFIP/H_2_0 (1:9, v/v), (d) NDIAPY in HFIP/H_2_0 (1:9, v/v).


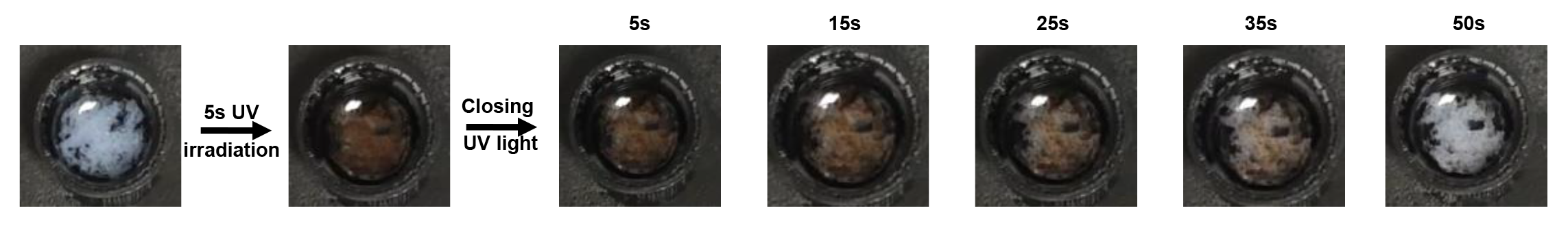


**Figure S16.** Time-dependent macrophotograph of LPF-NDIAPY in HFIP/ H_2_O_nitrogen_ (1:9, v/v) after 5s UV irradiation.


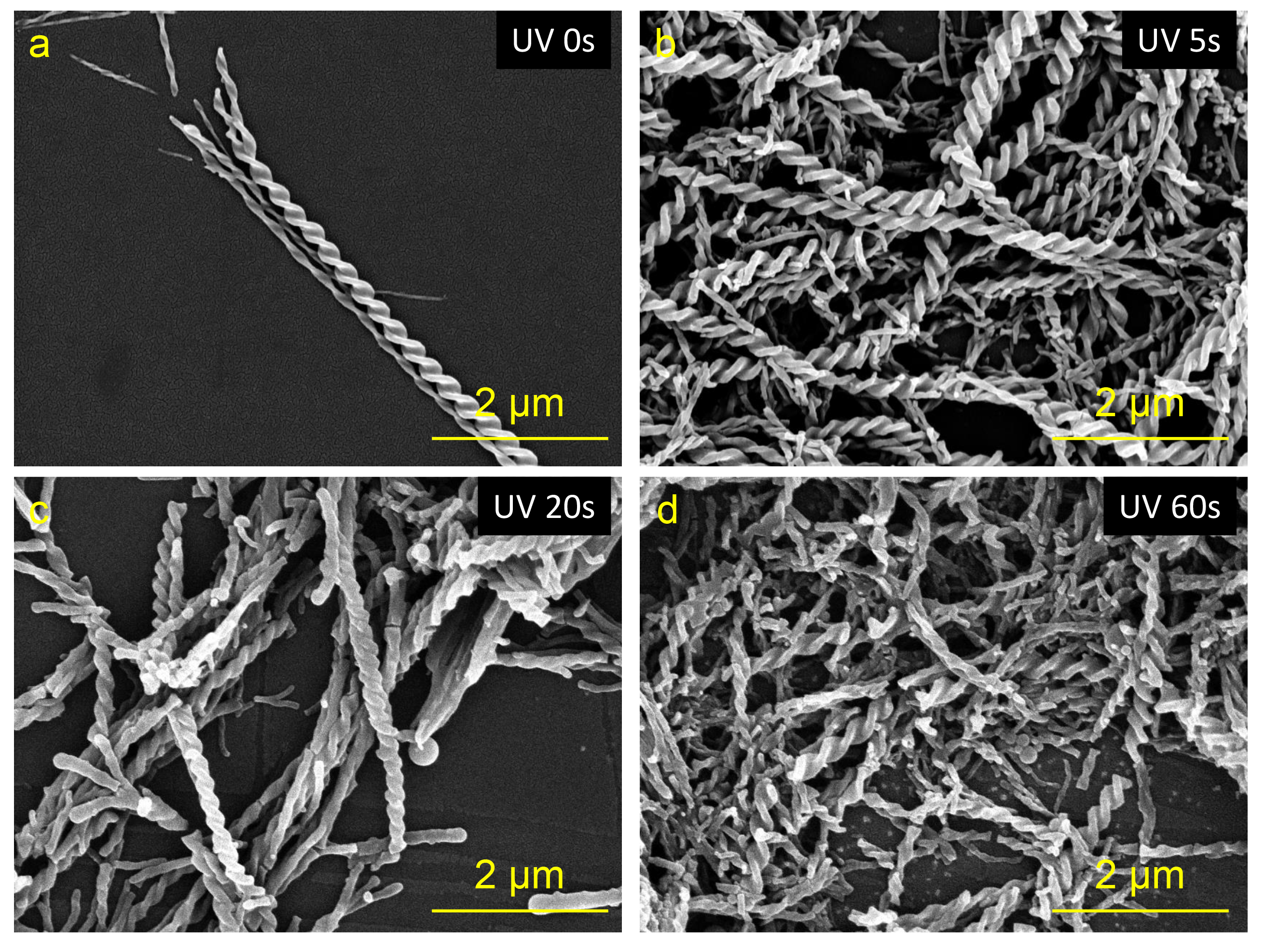


**Figure S17.** SEM images of LFP-NDIAPY after UV irradiation in low magnification for (a) 0s, (b) 5s, (c) 20s, (d) 60s.


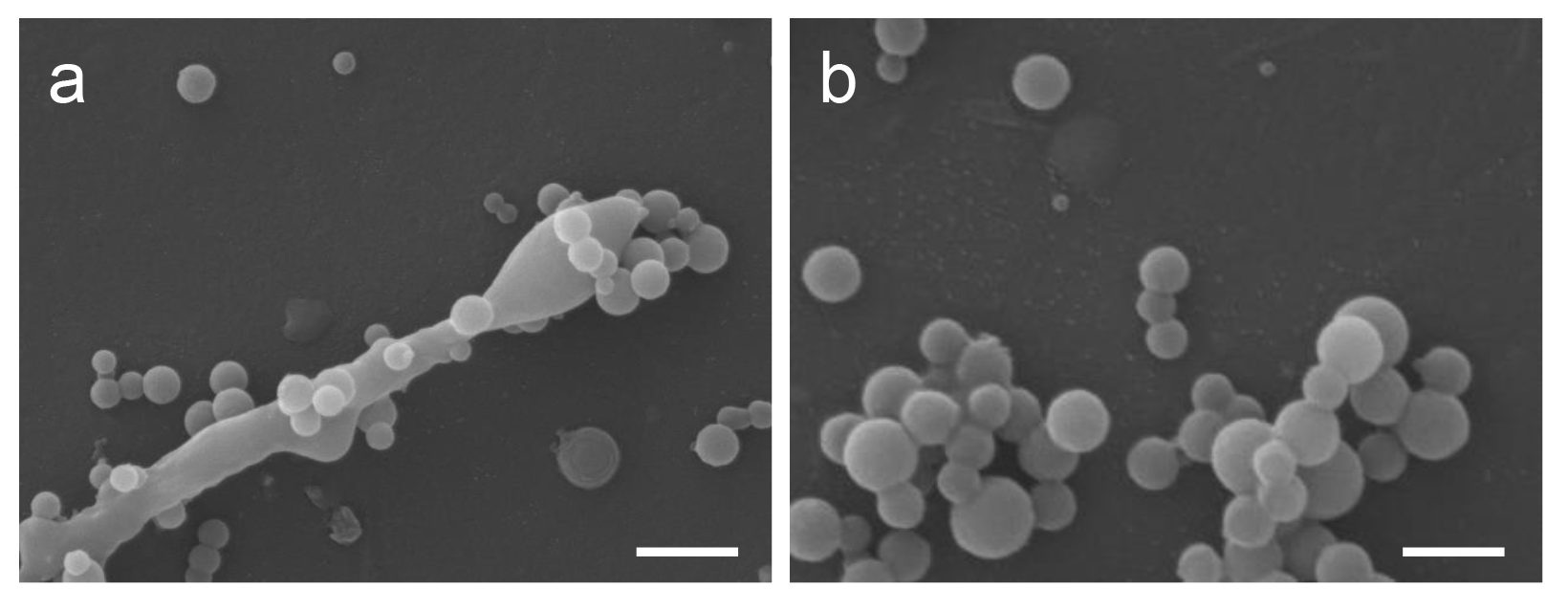


**Figure S18.** SEM images of LFP-NDIAPY for (a) ~1 min after 20s UV irradiation, (b) ~1 min after 60s UV irradiation. Scale bar: 1 μm for a, 500 nm for b.

**
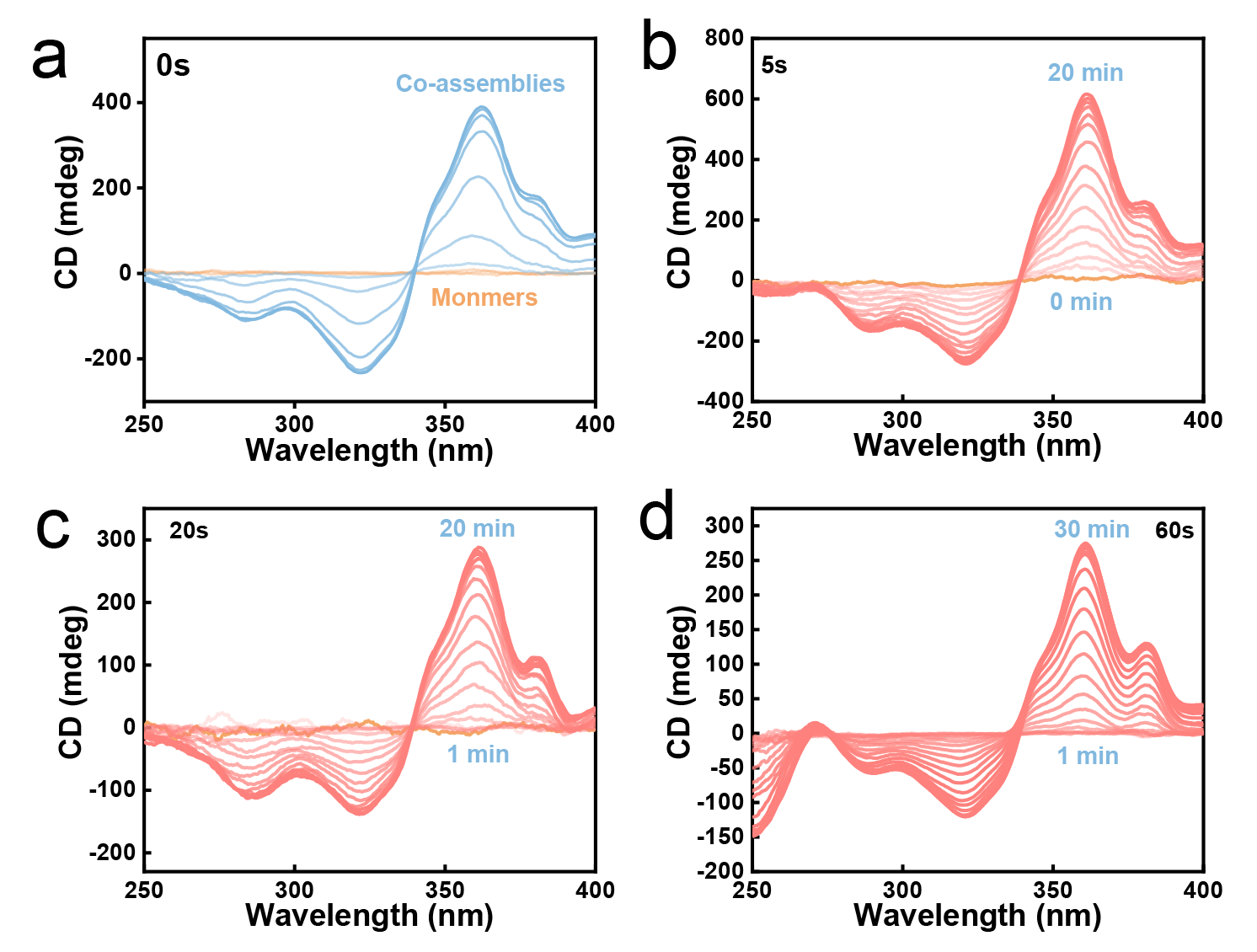
**

**Figure S19.** Time dependent CD spectra of (a) LPF-NDIAY, (b) after 5s UV irradiation, (c) after 20s UV irradiation, (d) after 60s UV irradiation.


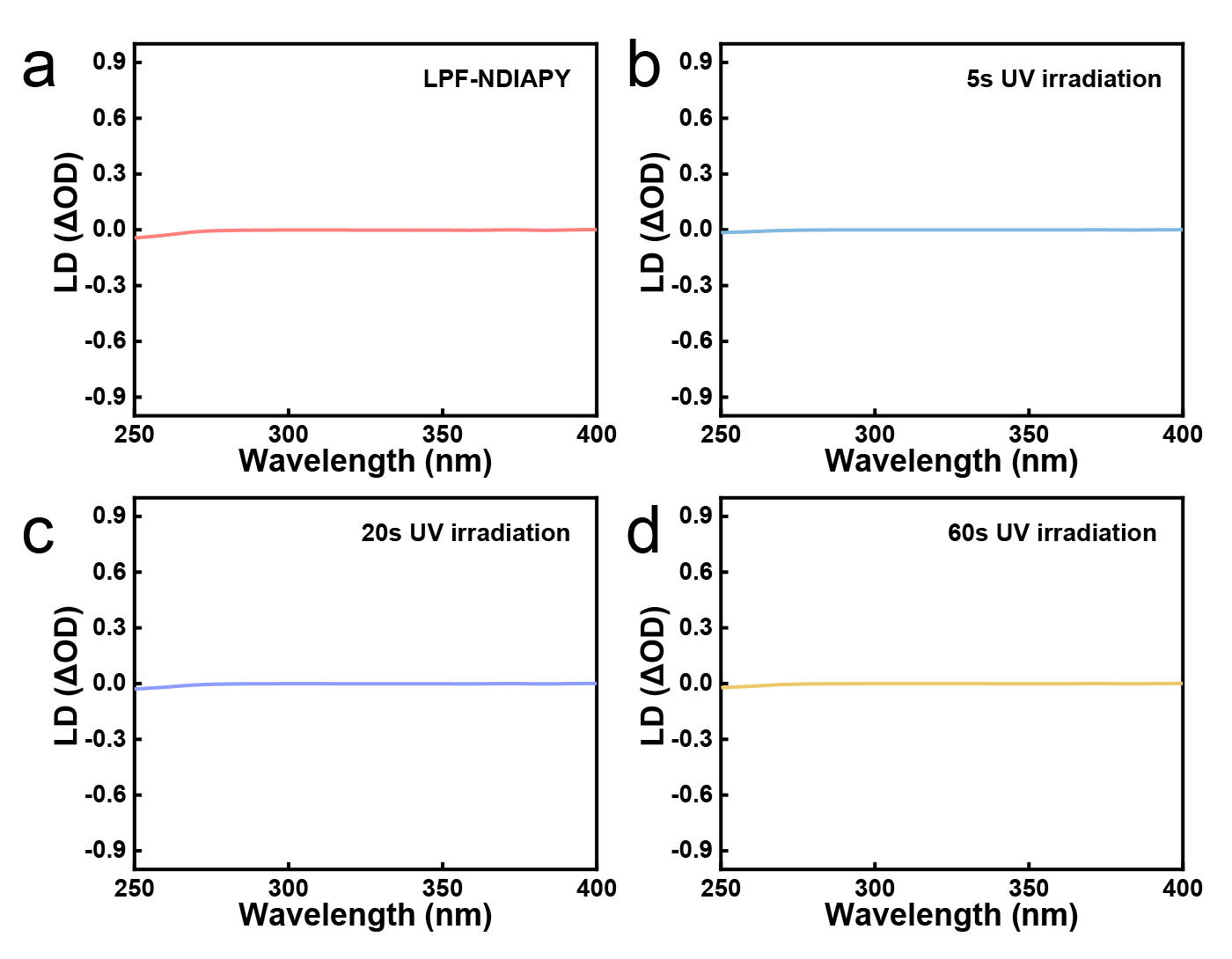


**Figure S20.** LD spectra of (a) LPF-NDIAY, (b) after 5s UV irradiation, (c) after 20s UV irradiation, (d) after 60s UV irradiation.


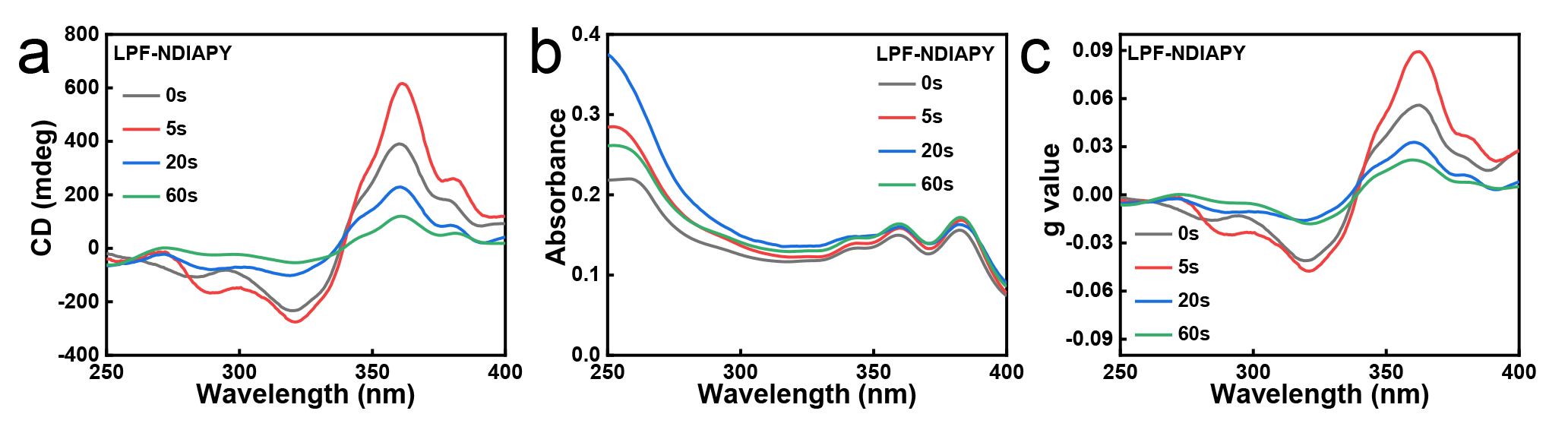


**Figure S21.** (a) CD, (b) UV, (c) g factor spectra of LFP-NDIAPY after 0s/5s/20/60s UV irradiation.


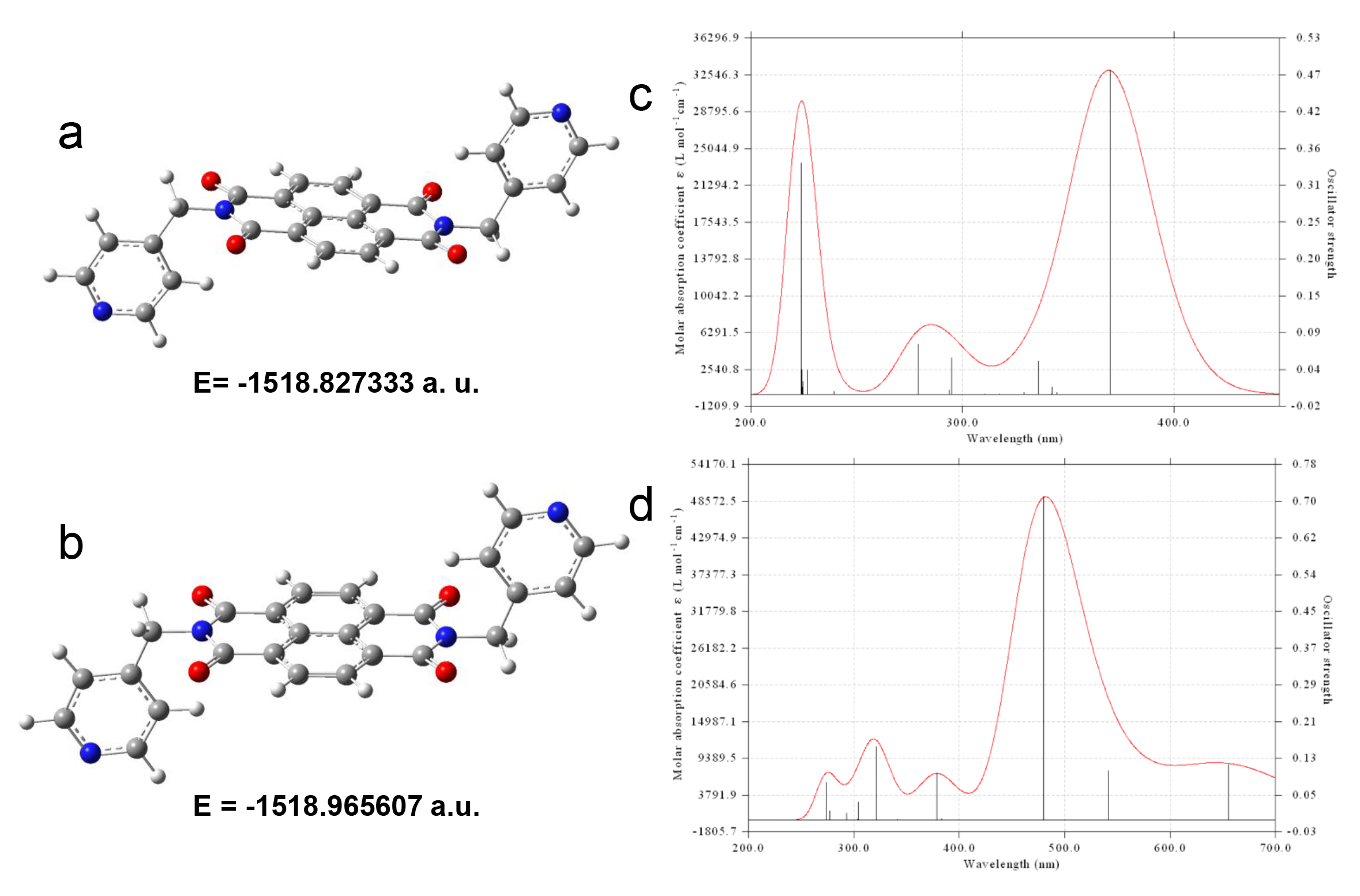


**Figure S22.** The optimized structure of (a) NDIAPY and (b) NDIAPY radical anion

by DFT calculations. The calculated UV spectra of (c) NDIAPY and (d) NDIAPY

radical anion by TD-DFT calculations.

**
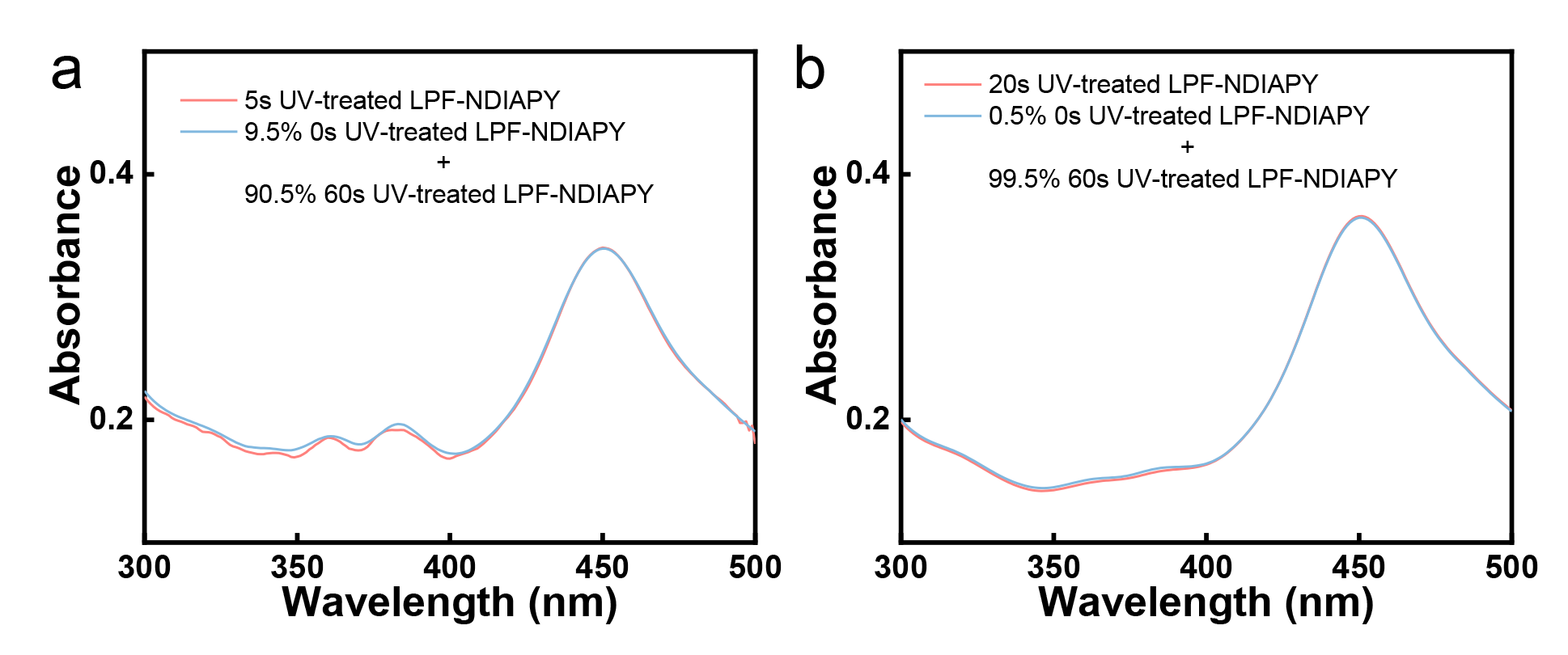
**

**Figure S23.**  (a) UV spectra of 5s UV-treated LPF-N DIAPY and 9.5% 0s UV-treated LPF-NDIAPY+ 90.5% 60s UV-treated LPF-NDIAPY, (b) UV spectra of UV spectra of 20s UV-treated LPF-N DIAPY and 0.5% 0s UV-treated LPF-NDIAPY+ 99.5% 60s UV-treated LPF-NDIAPY.


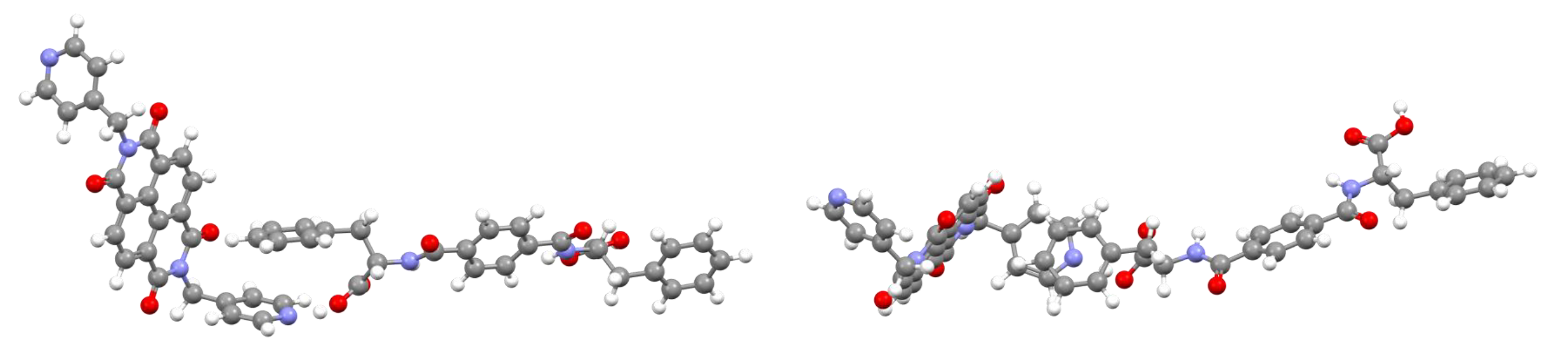


**Figure S24.** The different views of optimized structure for mode A.


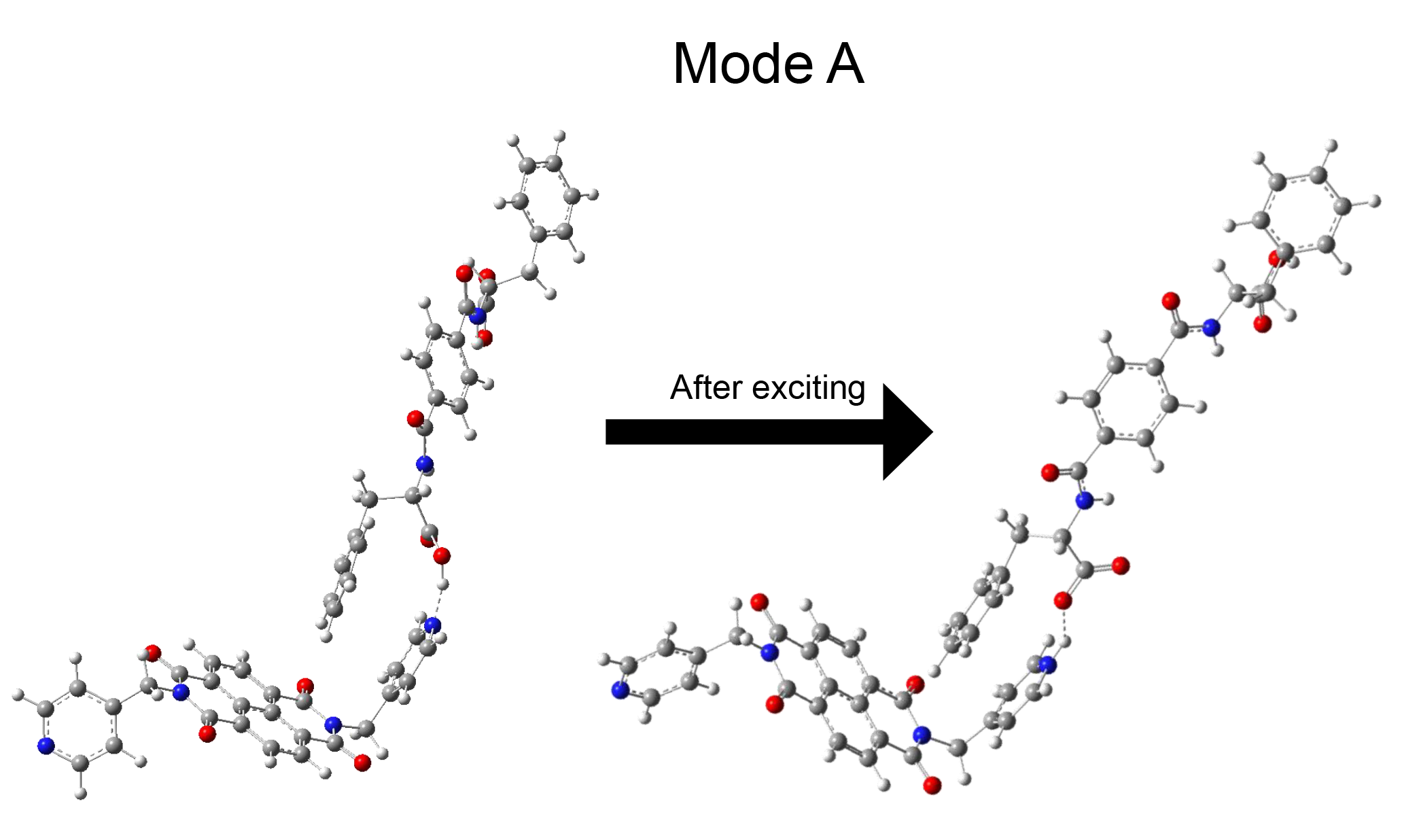


**Figure S25.** The optimized LPF-NDIAPY dimer based on the hydrogen bond between

carboxylic group and pyridine and its singlet excited-state.


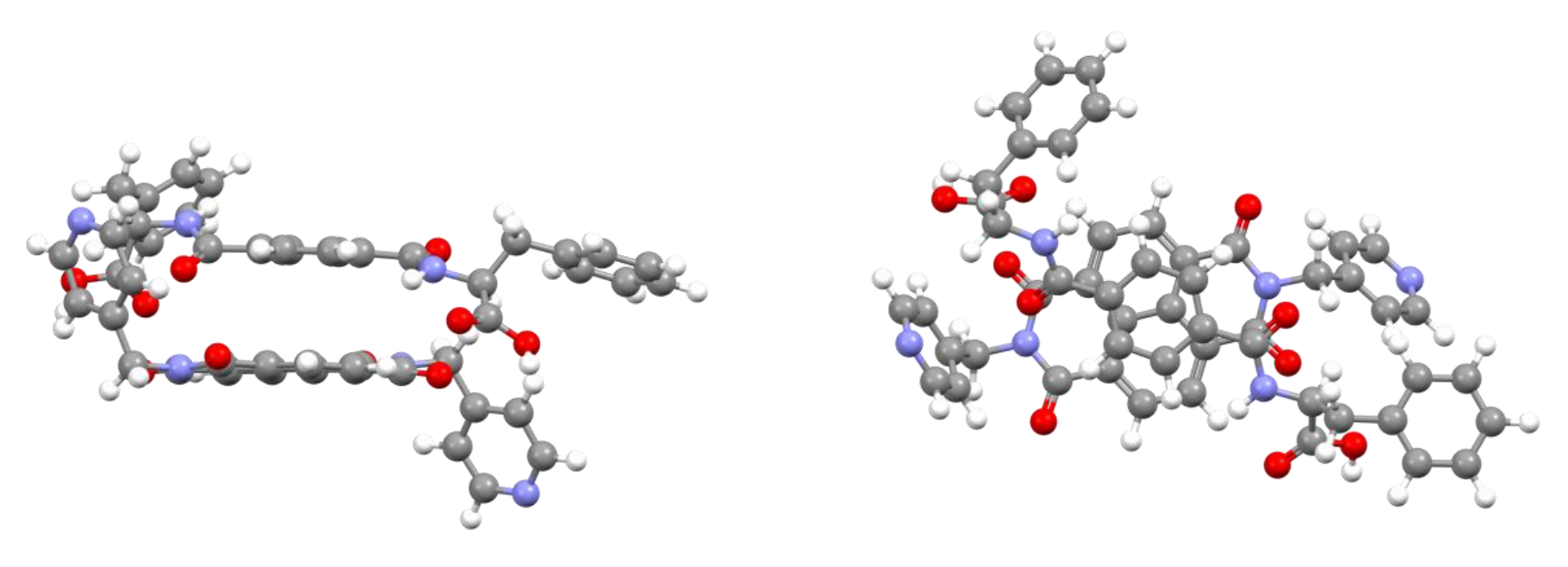


**Figure S26.** The different views of optimized structure for mode B.


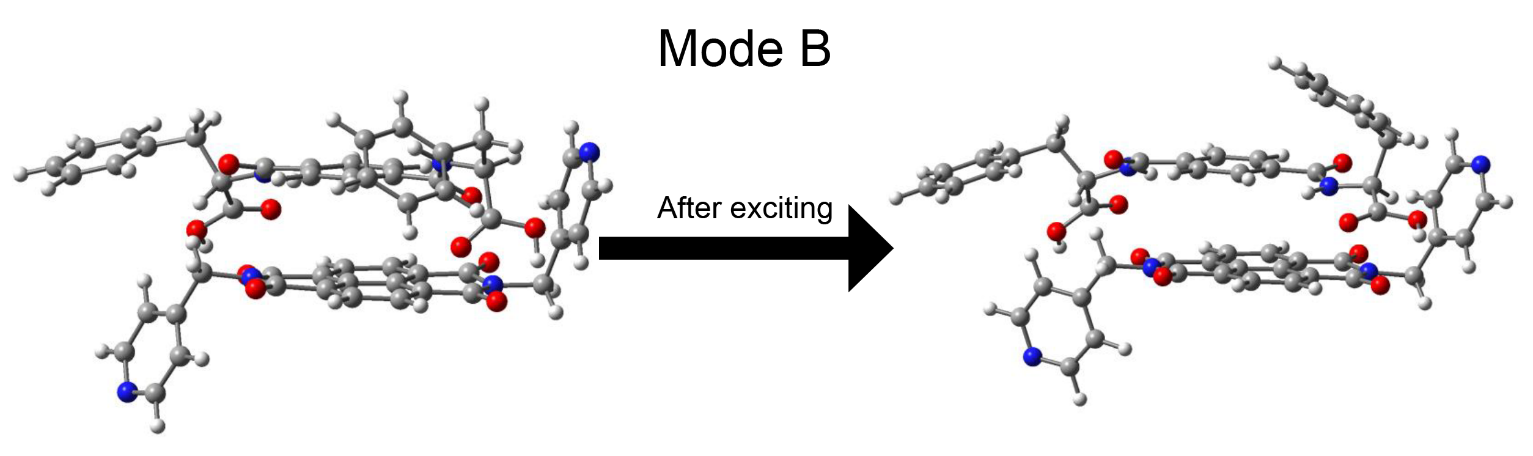


**Figure S27.** The optimized LPF-NDIAPY dimer based on the close contact between

phenyl core and NDI core and its singlet excited-state.


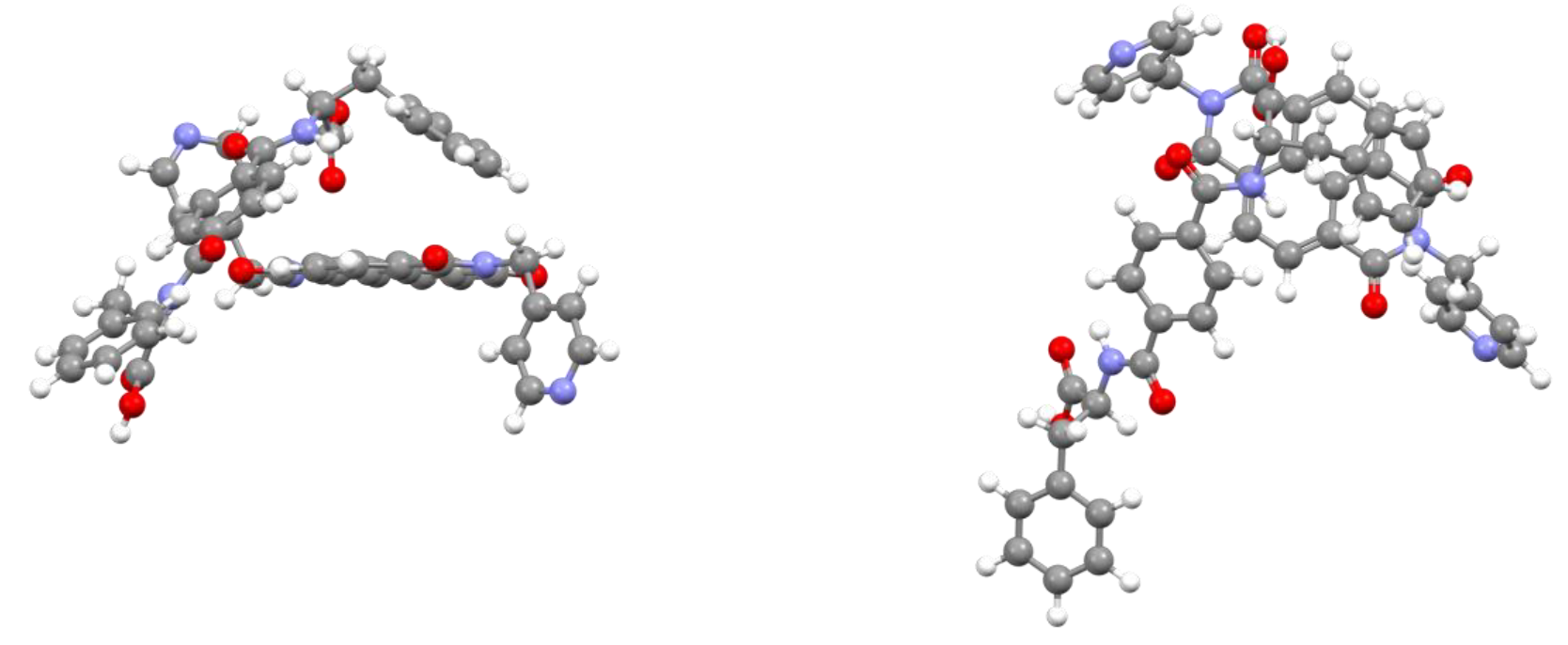


**Figure S28.**The different views of optimized structure for mode C.


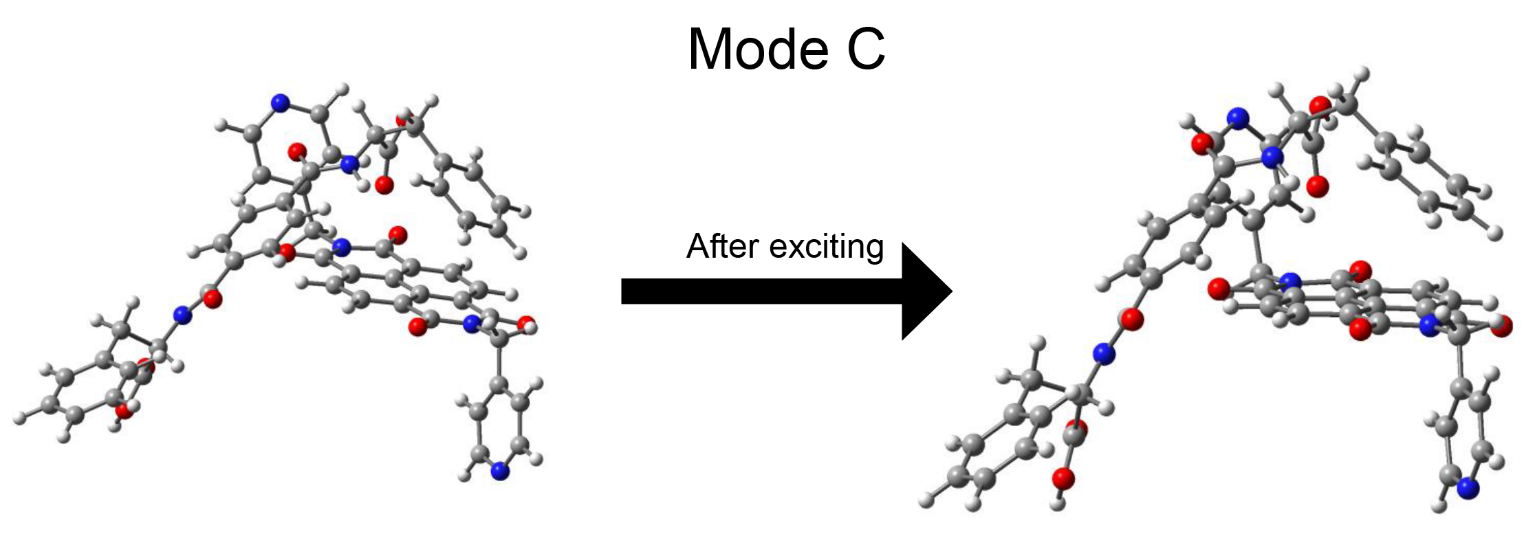


**Figure S29.** The optimized LPF-NDIAPY dimer based on the close contact between

phe group and NDI core and its singlet excited-state.


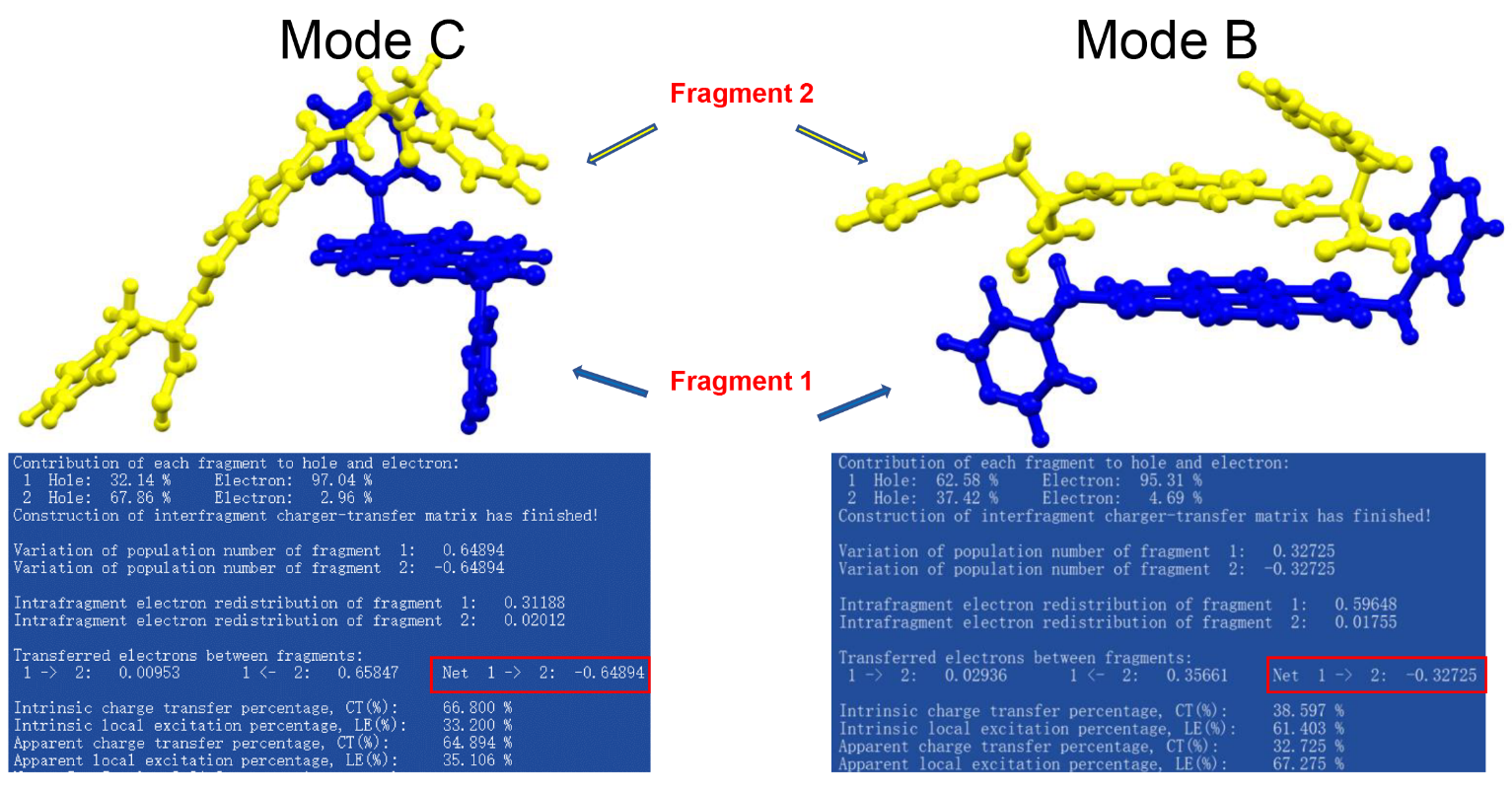


**Figure S30.** The net transferred electrons from LPF to NDIAPY based on mode B and

mode C.


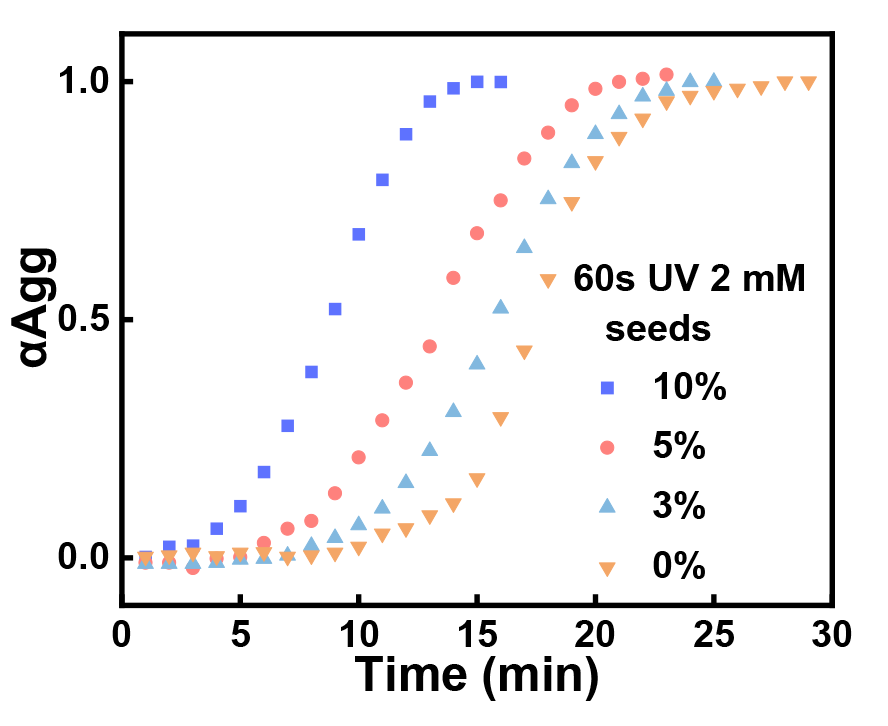


**Figure S31.** Time dependent CD signals at 361 nm of LFP-NDIAPY from 60s UV-

treated samples with different ratios of seeds. 0%, 3%, 5%, 10%.


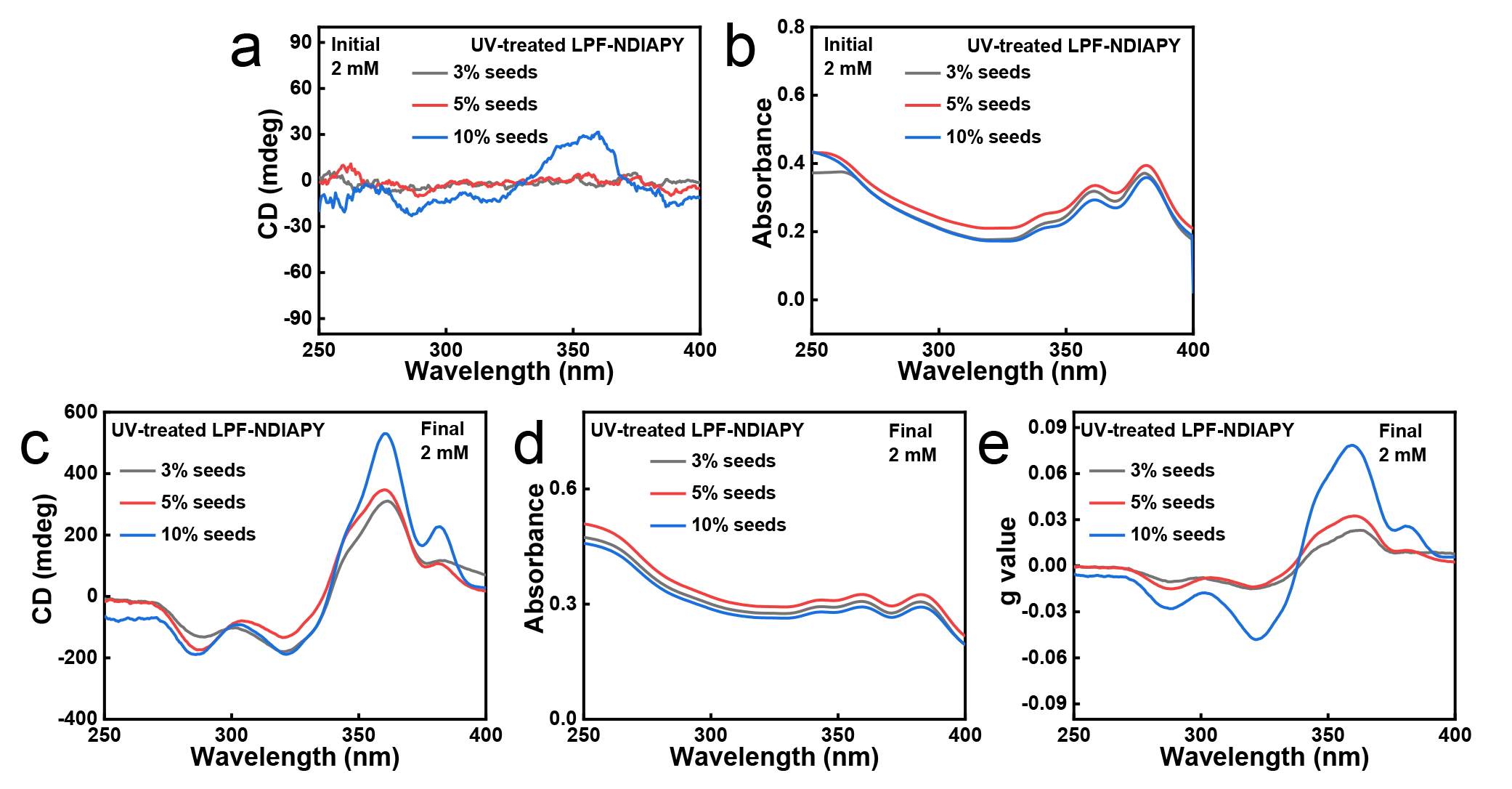


**Figure S32.** (a) Initial CD, (b) Initial UV, (c) Final CD, (d) Final UV, (e) g factor spectra of UV-treated LFP-NDIAPY with 3%/5%/10% seeds.


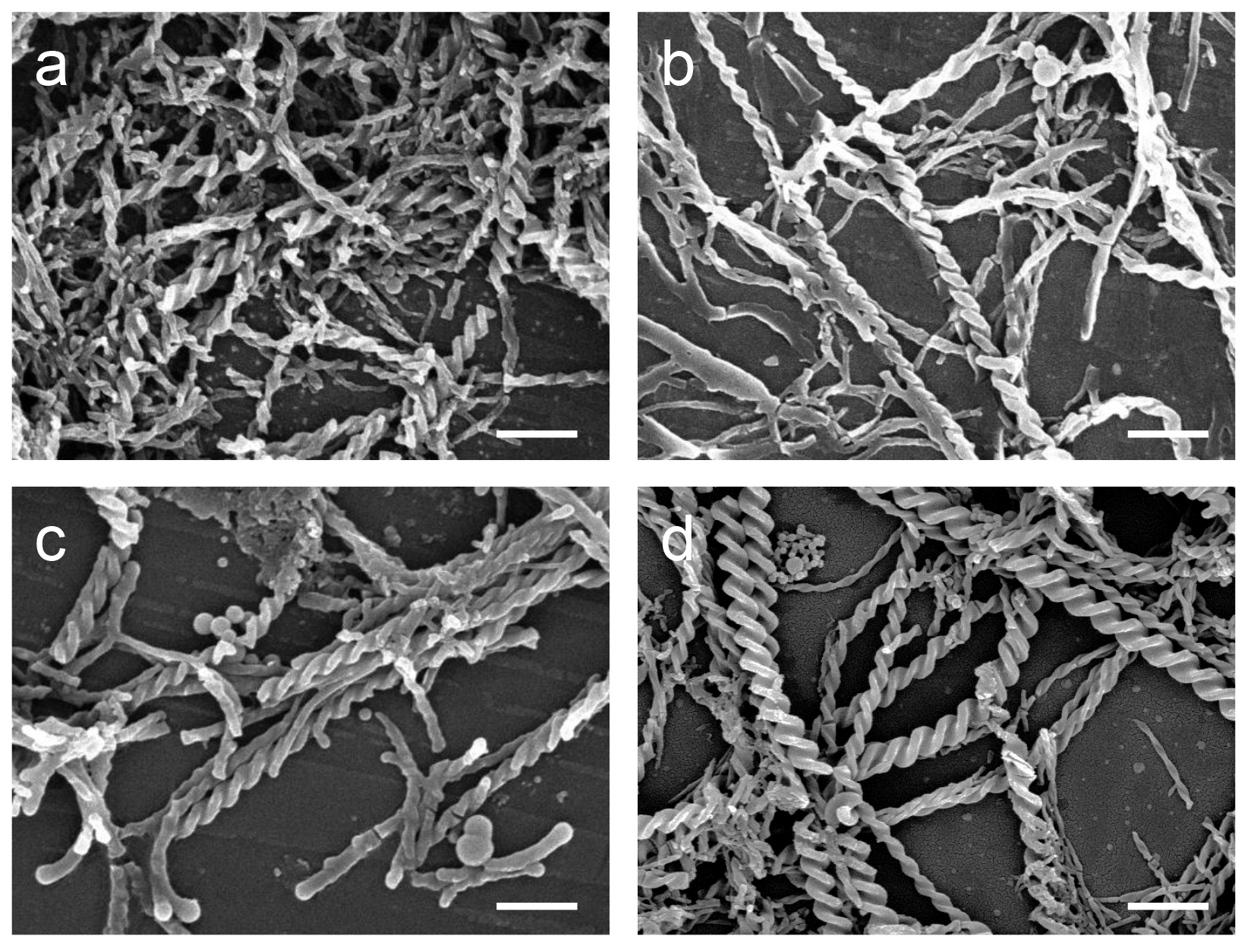


**Figure S33.** SEM images of LFP-NDIAPY from 60s UV-treated samples with different ratios of seeds. (a) 0%, (b) 3%, (c) 5%, (d) 10%. Scale bar: 1 μm.


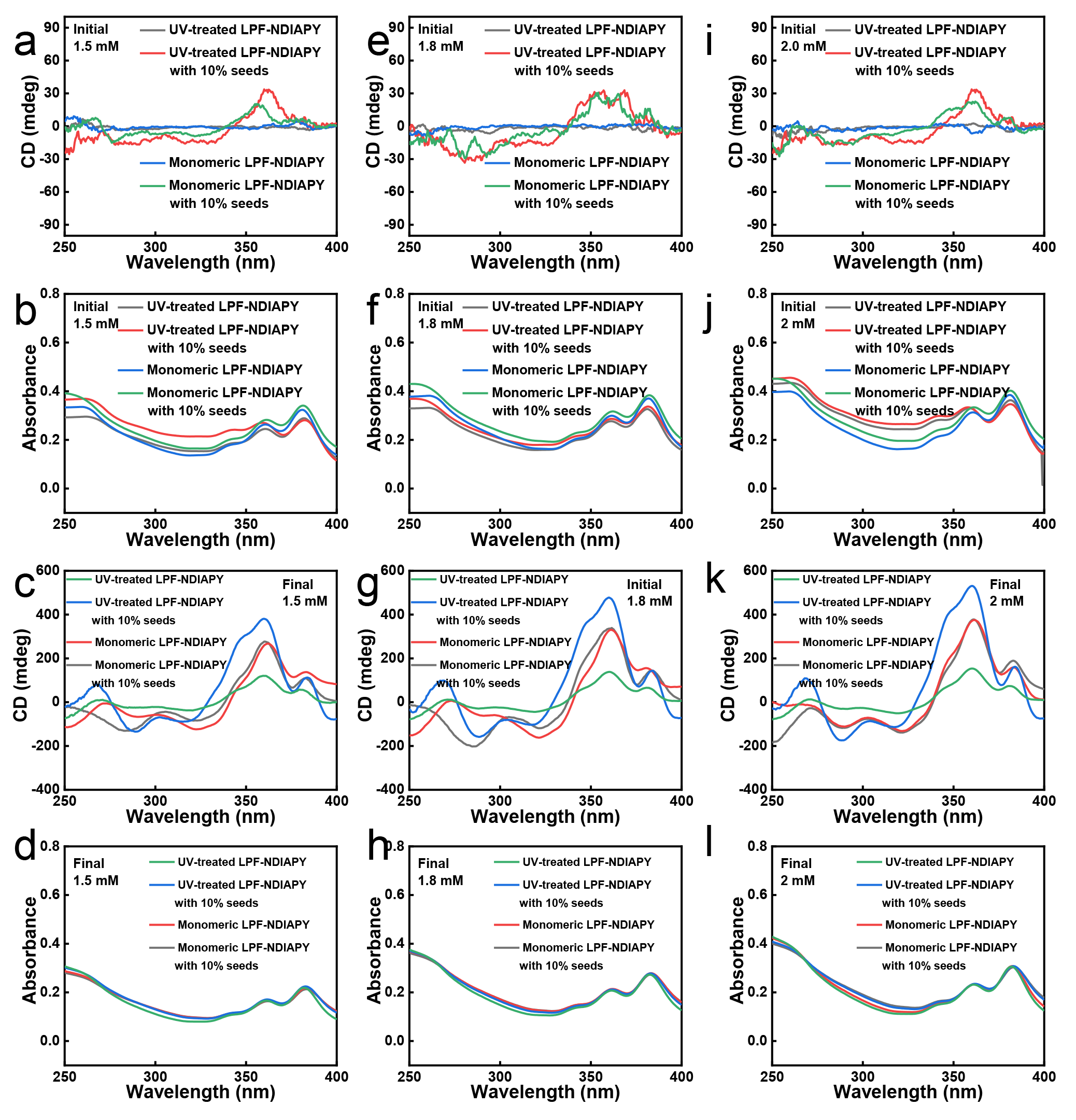


**Figure S34.** (a) Initial CD (1.5 mM), (b) Initial UV (1.5 mM), (c) Final CD (1.5 mM), (d) Final UV (1.5 mM), (e) Initial CD (1.8 mM), (f) Initial UV (1.8 mM), (g) Final CD (1.8 mM), (h) Final UV (1.8 mM), (i) Initial CD (2 mM), (j) Initial UV (2 mM), (k) Final CD (2 mM), (l) Final UV (2 mM) spectra of UV-treated LFP-NDIAPY, UV-treated LFP-NDIAPY with 10% seeds, monomeric LPF-NDIAPY, monomeric LPF-NDIAPY with 10% seeds.


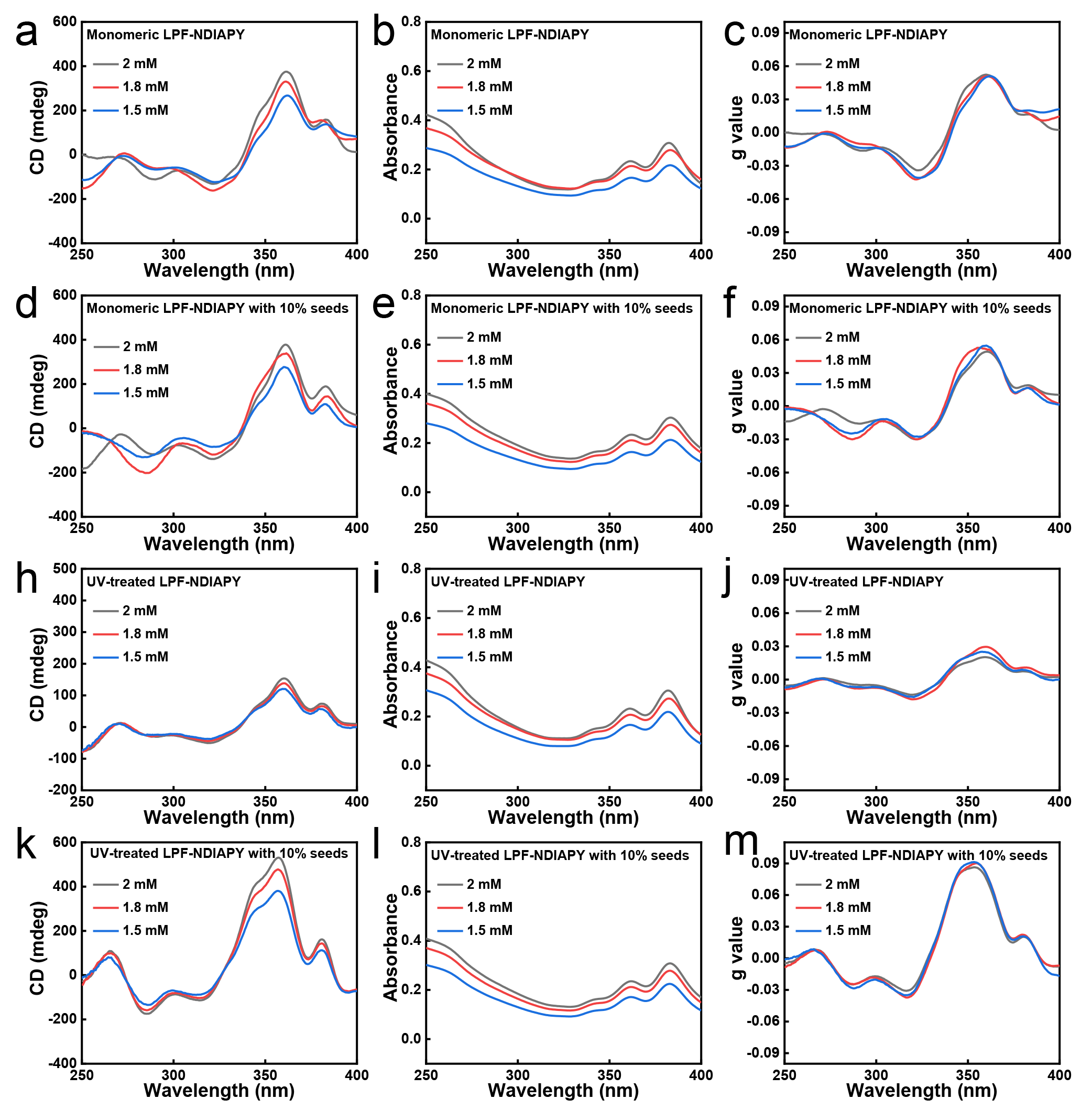


**Figure S35.** (a) CD, (b) UV, (c) g factor spectra of monomeric LFP-NDIAPY. (d) CD, (e) UV, (f) g factor spectra of monomeric LFP-NDIAPY with 10% seeds. (h) CD, (i) UV, (j) g factor spectra of UV-treated LFP-NDIAPY. (k) CD, (l) UV, (m) g factor spectra of UV-treated LFP-NDIAPY with 10% seeds.

**
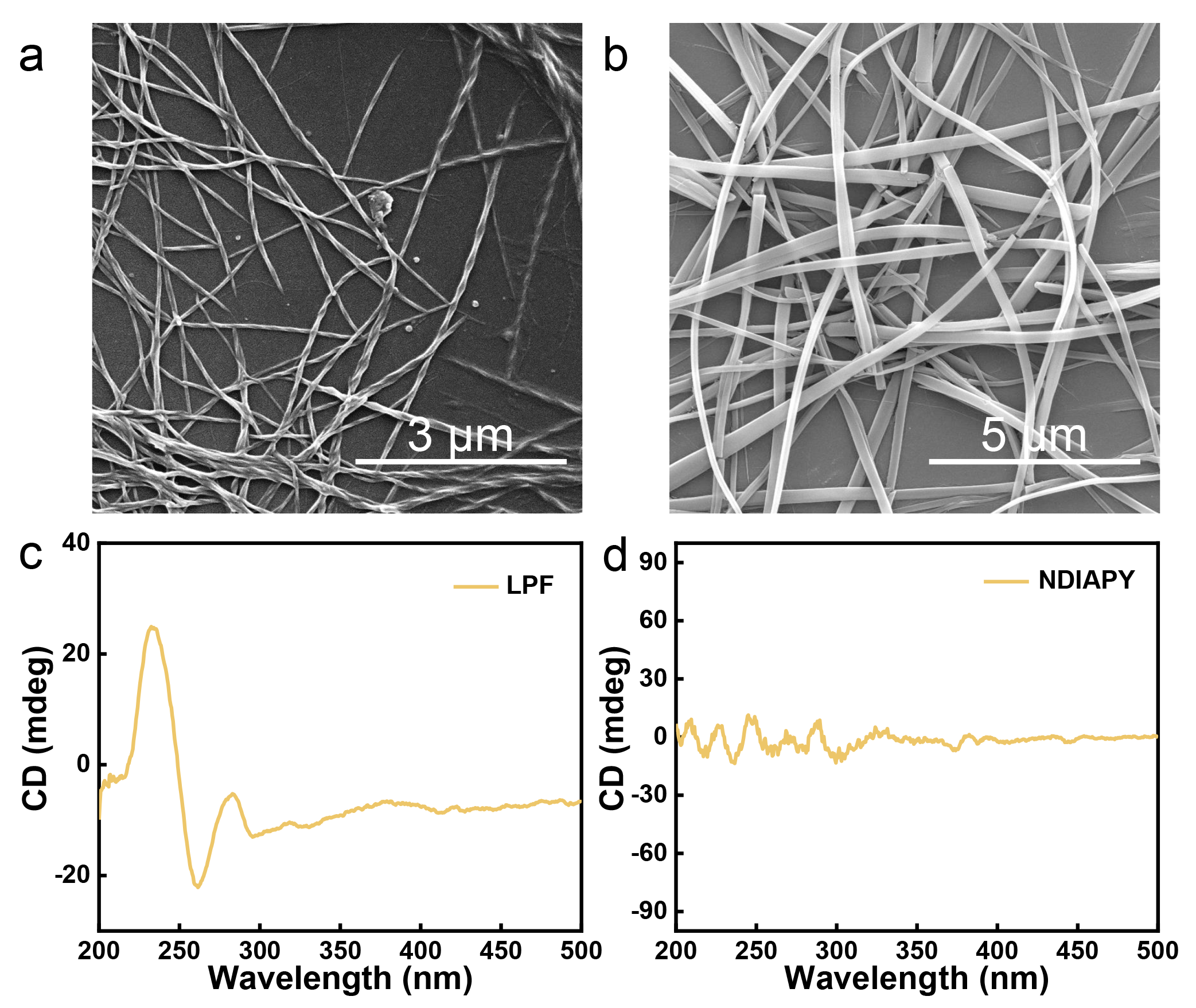
**

**Figure S36.** (a, c) SEM image and CD spectra of LPF. Scale bar: 3 μm. (b, d) SEM image and CD spectra of NDIAPY. Scale bar: 5 μm.


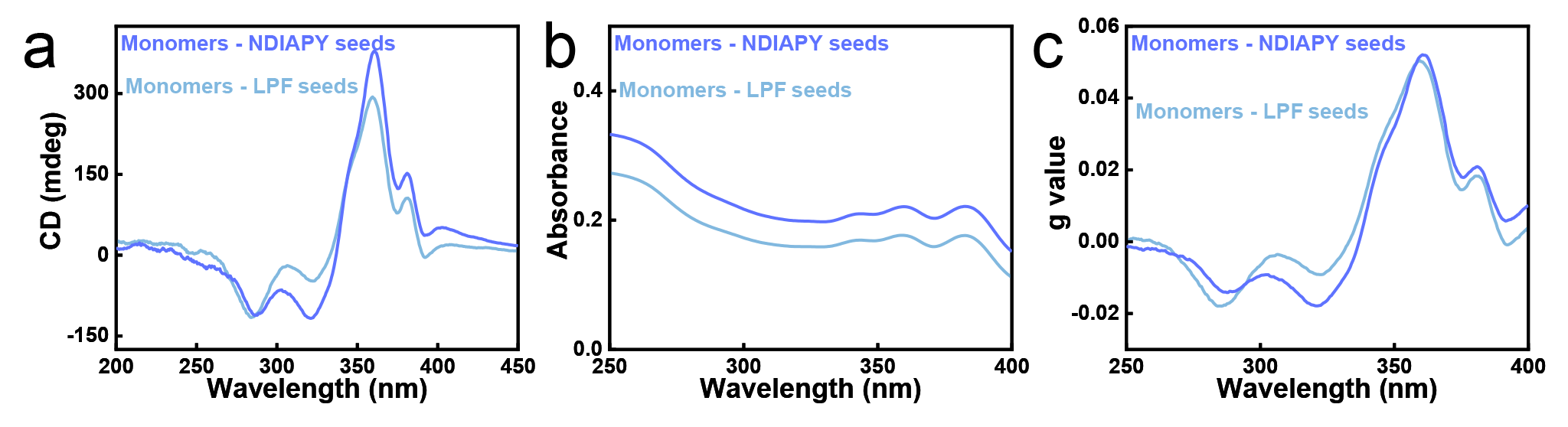


**Figure S37.** (a) CD, (b) UV, (c) g factor spectra of monomeric LFP-NDIAPY with NDIAPY seeds or LPF seeds.


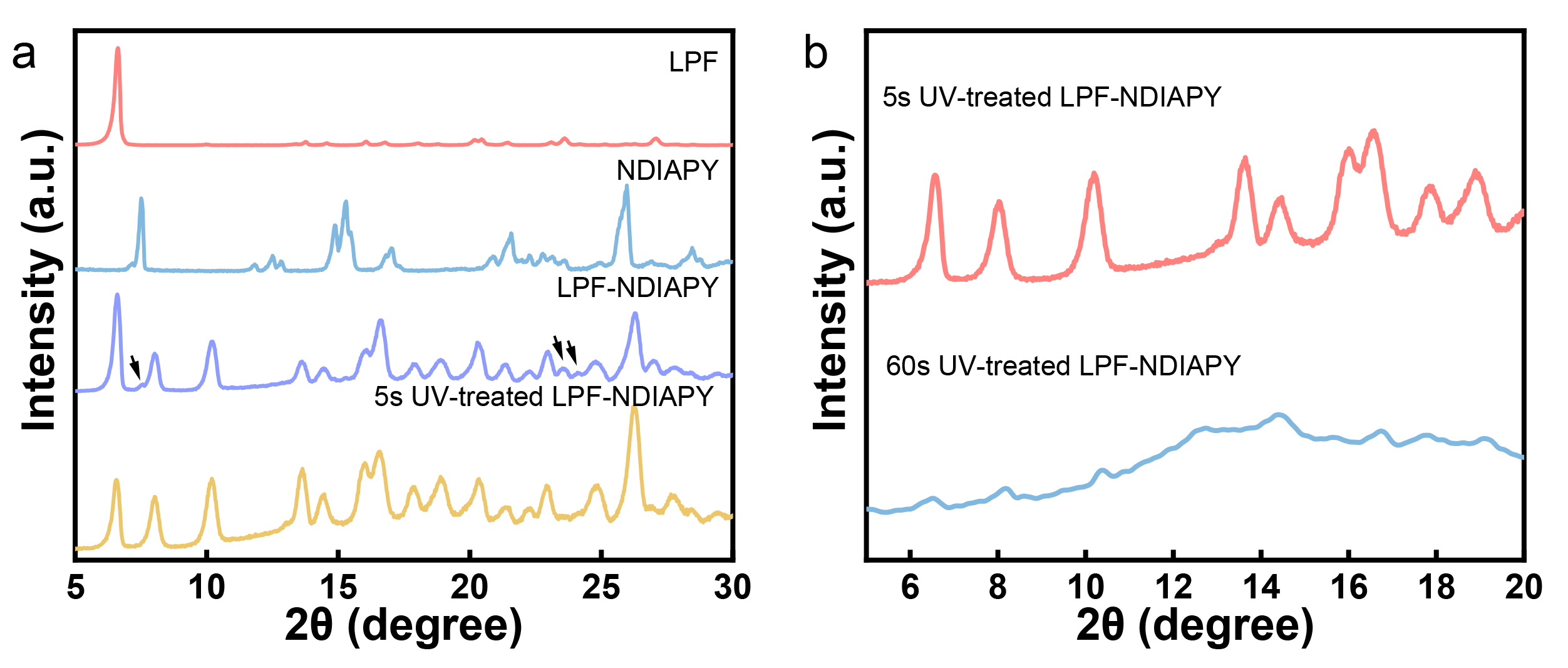


**Figure S38.** (a) XRD patterns of LPF, NDIAPY, LPF-NDIAPY, 5s UV-treated LPF-NDIAPY. (b) 5s UV-treated LPF-NDIAPY and 60s UV-treated LPF-NDIAPY.

# References

[1] Gaussian 16, Revision A.03, M.J. Frisch, G.W. Trucks, H.B. Schlegel, G.E. Scuseria, M.A. Robb, J.R. Cheeseman, G. Scalmani, V. Barone, G.A. Petersson, H. Nakatsuji, X. Li, M. Caricato, A.V. Marenich, J. Bloino, B.G. Janesko, R. Gomperts, B. Mennucci, H.P. Hratchian, J.V. Ortiz, A.F. Izmaylov, J.L. Sonnenberg, D. Williams-Young, F. Ding, F. Lipparini, F. Egidi, J. Goings, B. Peng, A. Petrone, T. Henderson, D. Ranasinghe, V.G. Zakrzewski, J. Gao, N. Rega, G. Zheng, W. Liang, M. Hada, M. Ehara, K. Toyota, R. Fukuda, J. Hasegawa, M. Ishida, T. Nakajima, Y. Honda, O. Kitao, H. Nakai, T. Vreven, K. Throssell, J.A. Montgomery, Jr., J.E. Peralta, F. Ogliaro, M.J. Bearpark, J.J. Heyd, E.N. Brothers, K.N. Kudin, V.N. Staroverov, T.A. Keith, R. Kobayashi, J. Normand, K. Raghavachari, A.P. Rendell, J.C. Burant, S.S. Iyengar, J. Tomasi, M. Cossi, J.M. Millam, M. Klene, C. Adamo, R. Cammi, J.W. Ochterski, R.L. Martin, K. Morokuma, O. Farkas, J.B. Foresman, and D.J. Fox, Gaussian, Inc., Wallingford CT, 2016.

[2] S. Grimme, J. Antony, S. Ehrlich, H. Krieg, J. Chem. Phys. 2010, 132, 154104.
